# A single-chain antibody generation system yielding CAR-T cells with superior antitumor function

**DOI:** 10.1038/s42003-021-01791-1

**Published:** 2021-03-02

**Authors:** Toshiki Ochi, Masaki Maruta, Kazushi Tanimoto, Fumitake Kondo, Toshihiro Yamamoto, Mie Kurata, Hiroshi Fujiwara, Junya Masumoto, Katsuto Takenaka, Masaki Yasukawa

**Affiliations:** 1 Department of Hematology, Clinical Immunology, and Infectious Diseases, Ehime University Graduate School of Medicine, Toon, Ehime Japan; 2 Division of Immune Regulation, Proteo-Science Center, Ehime University, Toon, Ehime Japan; 3 Department of Analytical Pathology, Ehime University Graduate School of Medicine, Toon, Ehime Japan; 4 Division of Pathology, Proteo-Science Center, Ehime University, Toon, Ehime Japan; 5 Department of Personalized Cancer Immunotherapy, Mie University Graduate School of Medicine, Tsu, Mie Japan; 6 Ehime Prefectural University of Health Sciences, Tobe, Ehime Japan

**Keywords:** Immunotherapy, Translational immunology

## Abstract

Cancer immunotherapy using T cells redirected with chimeric antigen receptor (CAR) has shown a lot of promise. We have established a single-chain antibody (scFv) generation system in which scFv library-expressing CAR-T cells can be screened appropriately based on their antitumor functions. A variable region library containing the variable and J regions of the human immunoglobulin light or heavy chain was fused with the variable region of a heavy or light chain encoded by an existing tumor-specific antibody to generate a new scFv library. Then, scFv library-expressing CAR-T cells were generated and stimulated with target cells to concentrate the antigen-specific population. Using this system, target-specific recognition of CAR-T cells appeared to be finely tuned by selecting a new variable region. Importantly, we have demonstrated that the newly optimized scFv-expressing CAR-T cells had better proliferation capacity and durable phenotypes, enabling superior reactivity against advanced tumors in vivo in comparison with the original CAR-T cells. Therefore, the optimization of an scFv is needed to maximize the in vivo antitumor functions of CAR-T cells. This system may allow us to adjust an immunological synapse formed by an scFv expressed by CAR-T cells and a target antigen, representing an ideal form of CAR-T-cell immunotherapy.

## Abstract (web summary)

With the aim of optimizing CAR-T cells for immunotherapy, Ochi et al. have established a system to generate and screen a library of CAR-T cells expressing different immunoglobulin variable regions. They show that this approach can enhance anti-tumour activities of CAR-T cells in vivo.

## Introduction

Chimeric antigen receptor (CAR)-redirected T cells display antitumor reactivity, and clinical trials of CAR-T-cell therapy demonstrated prolonged survival in patients with refractory CD19-positive malignancies^1–8^. These clinical trials suggest that the expansion and persistence of CAR-T cells appear to be important to achieve durable clinical responses^9,10^. To optimize CAR signaling for activating and maintaining T-cell functions, a series of studies focusing on manipulating spacer sequences^11–13^, and the intracellular domains^14–21^ of a CAR construct have been performed. In contrast, optimization of variable regions in a single chain fragment variable (scFv) for CAR-T cells has yet to be studied well, even though an scFv plays an important role in the recognition of a target antigen. Basically, using conventional methods, identification of antitumor scFvs seems to require 10^8^–10^10^ library sources of variable regions derived from immunoglobulin^22–24^. According to the previous strategies, an scFv with high binding capacity to a target antigen is likely to be selected for a CAR construct, and then the reactivity of CAR-T cells expressing the scFv should be further assessed. Since the binding ability of scFvs may not be always paralleled with the ideal functions of CAR-T cells, each scFv should be tested whether it would be suitable for CAR-T cells^25–29^. Therefore, optimization of variable regions of an scFv for CAR-T cells is a labor-consuming work and still appears to be uncertain. To address this concern, the development of a new system in which an scFv library is able to be both rapidly and accurately screened based on the antitumor functions of CAR-T cells would be expected to generate optimized scFv-expressing CAR-T cells with superior antitumor effects.

On the basis of previous phage display studies^23,30^, we have hypothesized that an scFv library combined an immunoglobulin variable region library of human peripheral blood B cells with a variable region derived from an existing antitumor antibody as a bait enables us to generate scFv library-expressing CAR-T cells. In this study, we have established a T-cell-based scFv generation system by exploiting CAR-library T cells. Second generation (CD28ζ) CAR-library T cells were stimulated with target cells to concentrate CAR-T cells with sufficient antitumor functions, then new scFvs were identified. We have confirmed superior in vivo antitumor effects of new scFv-optimized CAR-T cells against advanced tumors and mass-forming tumors, suggesting the need of fine-tuned scFvs for further advancement of CAR-T-cell therapy.

## Results

### Generation of CAR-library T cells specific for HLA-A2/NY-ESO-1_157_

**Fig. 1:**
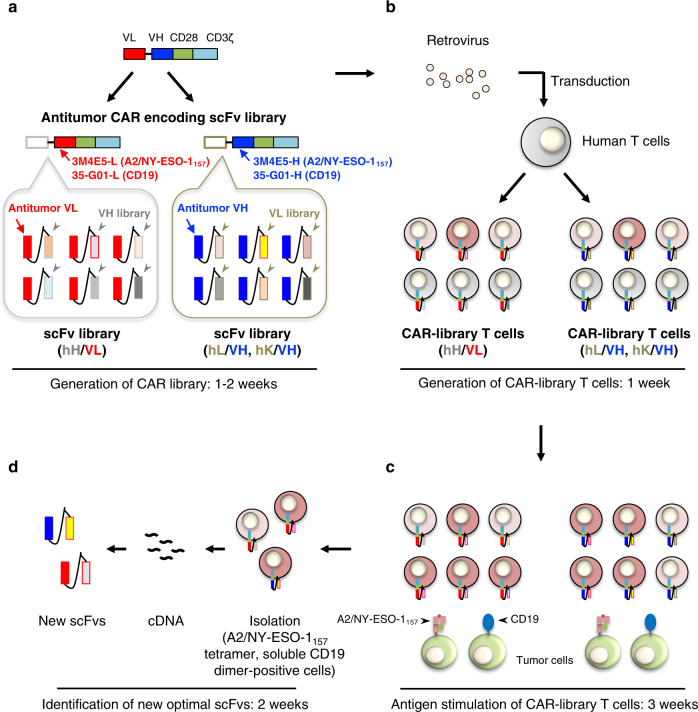
Identification of antitumor scFvs optimal for CAR-T cells using a T-cell-based scFv generation system. Each of the steps and times required for preparation of the CAR library, generation of CAR-library T cells, antigen stimulation of CAR-library T cells, and isolation of new scFvs are summarized. **a** Variable region libraries derived from the human immunoglobulin light chain (hL, hK) or heavy chain (hH) were prepared. These libraries were fused with the variable region of an existing antitumor antibody (VH, e.g. 3M4E5-H, 35-G01-H; VL, e.g. 3M4E5-L, 35-G01-L, indicated in blue and red, respectively) to generate antitumor scFv libraries. Then, a CAR library encoding the scFv library followed by the transmembrane/intracellular domains of a CAR (CD28ζ) construct was prepared. **b** A CAR library was retrovirally transduced into human peripheral blood T cells to generate antitumor CAR-library T cells. ScFv-optimized (dark red), scFv-suboptimal (light red), and non-reactive (gray) CAR-T cells can be included among CAR-library T cells. **c** Antitumor CAR-library T cells were stimulated with tumor cells expressing a target antigen, such as A2/NY-ESO-1_157_ and CD19, to concentrate the scFvs for delivery of activation signals to peripheral blood T cells via a CAR construct. **d** After isolation of antigen-specific CAR-T cells by flow cytometry using A2/NY-ESO-1_157_ tetramer and soluble CD19 dimer, cDNA was synthesized and the primary structures of new scFvs that would be optimal for CAR-T cells were determined.

**Fig. 2:**
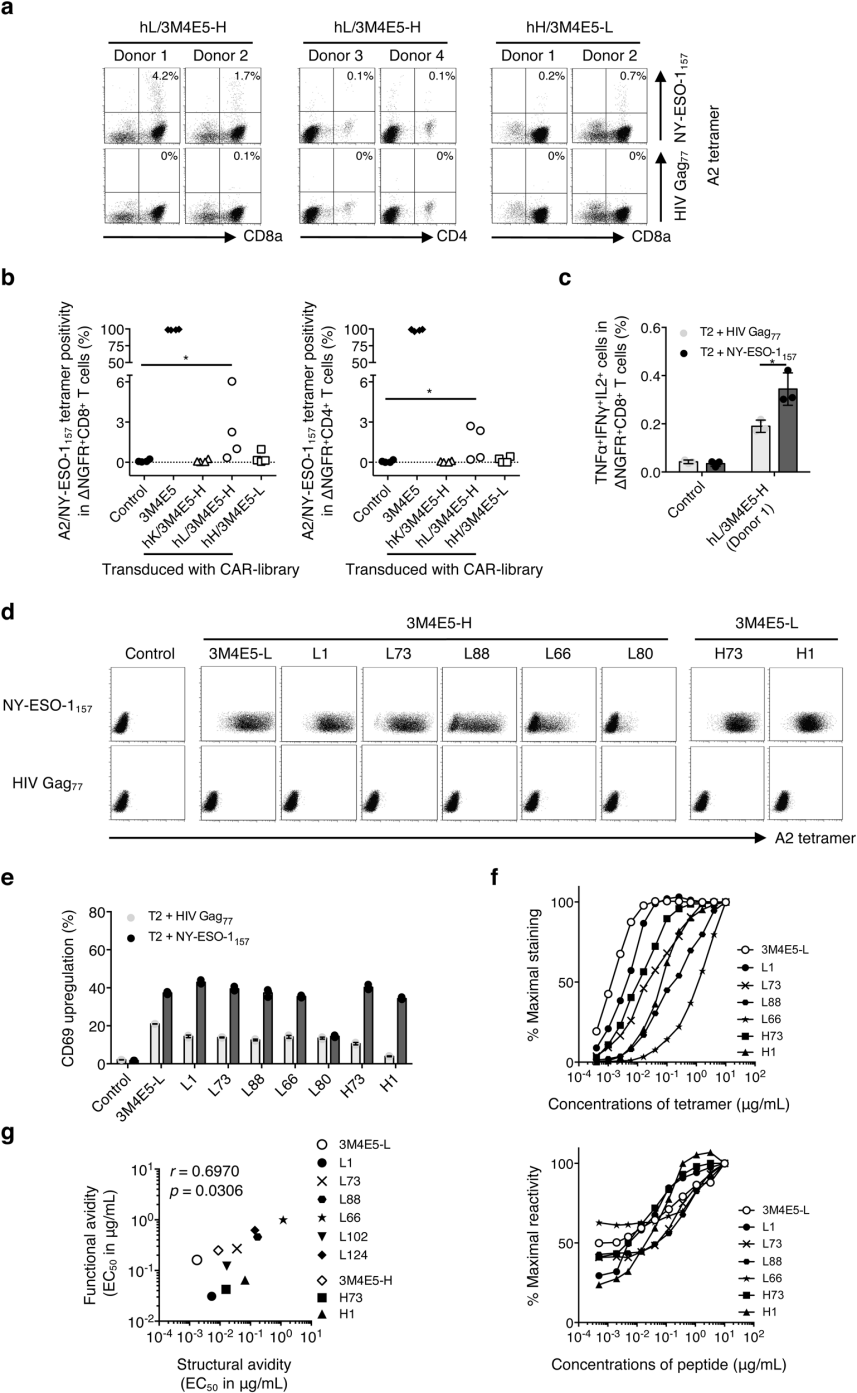
Target reactivity of CAR-T cells expressing new scFvs identified from A2/NY-ESO-1_157_ CAR-library T cells. **a** A2/NY-ESO-1_157_-specific scFv libraries possessing the variable and J regions of each human kappa chain (hK), lambda chain (hL) or heavy chain (hH) library derived from four different donors along with the A2/NY-ESO-1_157_-specific 3M4E5-H or 3M4E5-L, indicated as hK/3M4E5-H, hL/3M4E5-H, and hH/3M4E5-L, were individually generated. A second-generation CAR library encoding each A2/NY-ESO-1_157_-specific scFv library together with CD28ζ was transduced into peripheral blood T cells. A2/NY-ESO-1_157_ CAR-library T cells similarly stimulated three times were stained with 20 μg/mL A2/NY-ESO-1_157_ or A2/HIV Gag_77_ tetramer. Representative stainings of A2/NY-ESO-1_157_ CAR-library CD8^+^ T cells (hL/3M4E5-H and hH/3M4E5-L) derived from donors 1 and 2, and CD4^+^ T cells (hL/3M4E5-H) derived from donors 3 and 4 are shown. Each percentage of A2/NY-ESO-1_157_ tetramer-positive cells among CAR-library T cells is indicated. **b** A2/NY-ESO-1_157_ tetramer positivity in CAR-library CD8^+^ T cells and CD4^+^ T cells is summarized. Each dot represents each library source derived from the different donors. The original 3M4E5 CAR-T cells and control T cells were prepared as a positive and a negative control, respectively. Two-tailed Mann–Whitney test was performed to compare two different groups. *p < 0.05. **c** Control T cells, or hL/3M4E5-H CAR-library CD8^+^ T cells (donor 1) were incubated with T2 cells pulsed with 10 μg/mL NY-ESO-1_157_ peptide or HIV Gag_77_ peptide. Multiple cytokine production by these CAR-T cells was measured by intracellular cytokine staining via flow cytometry. TNFα^+^IFNγ^+^IL2^+^ cells in ΔNGFR^+^CD8^+^ T cells are shown. The experiments were performed in triplicate, and error bars show the SD. Welch’s *t* test (two-sided) was performed for comparison. *p < 0.05. **d**, **e** Newly identified A2/NY-ESO-1_157_ CARs were individually reconstituted in Jurkat 76 cells. Jurkat 76/CAR transfectants expressing each different VL (3M4E5-L, L1, L73, L88, L66, or L80) or VH (H73 or H1) chain paired with 3M4E5-H or 3M4E5-L were stained with 5 μg/mL A2/NY-ESO-1_157_ or A2/HIV Gag_77_ tetramer. Staining of control Jurkat 76 cells is also shown as a negative control (**d**). Jurkat 76/CAR transfectants were cocultured with T2 cells loaded with 10 μg/mL NY-ESO-1_157_ peptide or HIV Gag_77_ peptide. CD69 upregulation of Jurkat 76/CAR transfectants was measured by flow cytometry. The experiments were performed in triplicate, and error bars depict the SD (**e**). **f** Jurkat 76/CAR transfectants were stained with graded concentrations of A2/NY-ESO-1_157_ tetramer (top). They were also incubated with T2 cells pulsed with graded concentrations of A2/NY-ESO-1_157_ peptide (bottom). Percentage maximal staining of each indicated transfectant was calculated relative to the staining pattern of each transfectant with 10 μg/mL A2/NY-ESO-1_157_ tetramer as 100% (top). Percentage maximal reactivity of each transfectant was measured relative to the CD69 upregulation of each transfectant for 10 μg/mL NY-ESO-1_157_ peptide loaded onto T2 cells as 100% (bottom). **g** Structural avidity, shown as EC_50_ values in μg/mL, was calculated as the concentration of A2/NY-ESO-1_157_ tetramer required to achieve 50% of the maximal staining. Functional avidity, expressed as EC_50_ values in μg/mL, was estimated as the concentration of NY-ESO-1_157_ peptide required to obtain 50% of the maximal reactivity. Each dot represents each Jurkat 76/CAR transfectant. The nonparametric Spearman correlation coefficient was calculated. *r* and *p* values are shown.

First, we employed an antibody (clone 3M4E5) specific for an HLA-A*02:01/NY-ESO-1_157-165_ complex (A2/NY-ESO-1_157_) as a model^31–33^. We investigated whether A2/NY-ESO-1_157_-specific CAR-T cells show target reactivity. A2/NY-ESO-1_157_ light–heavy (LH) and heavy–light (HL) second generation (CD28ζ) CARs in which variable regions of the light (VL) and heavy (VH) chain were linked in order of L to H (3M4E5-LH), or vice versa (3M4E5-HL) were synthesized. Both 3M4E5-LH and 3M4E5-HL CAR-T cells recognized A2/NY-ESO-1_157_ (Supplementary Fig. 1). A variable region library (hL, hK, and hH) containing the variable and J regions at the N-terminus derived from immunoglobulin of four donors was individually fused with 3M4E5-H or 3M4E5-L at the C-terminus to generate an A2/NY-ESO-1_157_-specific scFv library (Fig. 1a). After confirming that the scFv library was heterogeneous by direct sequencing (Supplementary Fig. 2a), each A2/NY-ESO-1_157_-specific CAR library was generated and transduced into peripheral blood T cells (Fig. 1b). Transduction efficiency of CAR-library T cells showed around 30%, suggesting that it was expected that one CAR construct is expressed in one T cell^34^ (Supplementary Fig. 2b). Then, the CAR-library T cells were stimulated with K562-based antigen-presenting cells (APCs) expressing the target antigen (Fig. 1c). K562 cells expressing an HLA-A*02:01 together with CD80 and CD83 molecules were pulsed with NY-ESO-1_157_ peptide, and employed as APCs^35–37^. Before stimulation, an A2/NY-ESO-1_157_-specific population among CAR-library T cells was rarely detected by A2/NY-ESO-1_157_ tetramer (Supplementary Fig. 2c). In contrast, after three stimulations, A2/NY-ESO-1_157_-specific CAR-library (hL/3M4E5-H, hH/3M4E5-L) CD8^+^ T cells and CAR-library (hL/3M4E5-H) CD4^+^ T cells generated from different donors were successfully concentrated (Fig. 2a). A2/NY-ESO-1_157_ tetramer-positive cells were frequently detected among hL/3M4E5-H CAR-library T cells, and hL/3M4E5-H CAR-library CD8^+^ T cells containing a high A2/NY-ESO-1_157_-specific population produced multiple cytokines in response to stimulation with T2 cells pulsed with NY-ESO-1_157_ peptide (Fig. 2b, c). These results suggest that antigen-specific CAR-library T cells can be expanded by antigen stimulation, resulting in successful enrichment of scFvs which can deliver activation signals to peripheral blood T cells via a CAR construct.

### Various reactivities of CAR-T cells expressing new scFvs isolated from A2/NY-ESO-1_157_-specific CAR-library T cells

The A2/NY-ESO-1_157_ tetramer-positive cells among the CAR-library T cells that had been established were then isolated by flow cytometry. The sequence of each new scFv was determined from sorted CAR-T cells after cDNA generation (Fig. 1d). Representative new 8 VLs and 2 VHs paired with 3M4E5-H or 3M4E5-L were further investigated. The variable regions and amino acid sequences of their complementarity-determining region (CDR) 3 were heterogeneous (Supplementary Table 1). Second generation (CD28ζ) CAR constructs each containing a different scFv were individually reconstituted in Jurkat 76 cells to assess their reactivity against A2/NY-ESO-1_157_^38^. Note that ΔNGFR-positive cells were isolated, gated and analyzed to compare gene-modified cells similarly. Consequently, Jurkat 76/CAR transfectants recognized A2/NY-ESO-1_157_ to different degrees (Fig. 2d, e; Supplementary Fig. 3a, b). Then, structural avidities of representative Jurkat 76/CAR transfectants were measured using graded concentrations of A2/NY-ESO-1_157_ tetramer. Furthermore, functional avidities of these transfectants were also examined using T2 cells pulsed with graded concentrations of NY-ESO-1_157_ peptide. Jurkat 76/CAR transfectants showed a broad range of avidities to A2/NY-ESO-1_157_ (Fig. 2f; Supplementary Fig. 3c). The structural and functional avidities appeared to be positively correlated at high EC_50_ values, but did not appear to be correlated at low EC_50_ values (Fig. 2g; Supplementary Table 1). Interestingly, L52 CAR-transduced Jurkat 76 cells recognized T2 cells without extracellularly loaded peptides, and their reactivity against T2 cells was not significantly enhanced by adding NY-ESO-1_157_ or HIV Gag_77_ peptide when L52 was paired with 3M4E5-H (Supplementary Fig. 3d). These results suggest that target-specific reactivity of CAR-T cells is able to be changed by modulating a variable region of an scFv expressed by CAR-T cells.

### Target reactivity of A2/NY-ESO-1_157_-specific CAR-T cells expressing a fine-tuned scFv

**Fig. 3:**
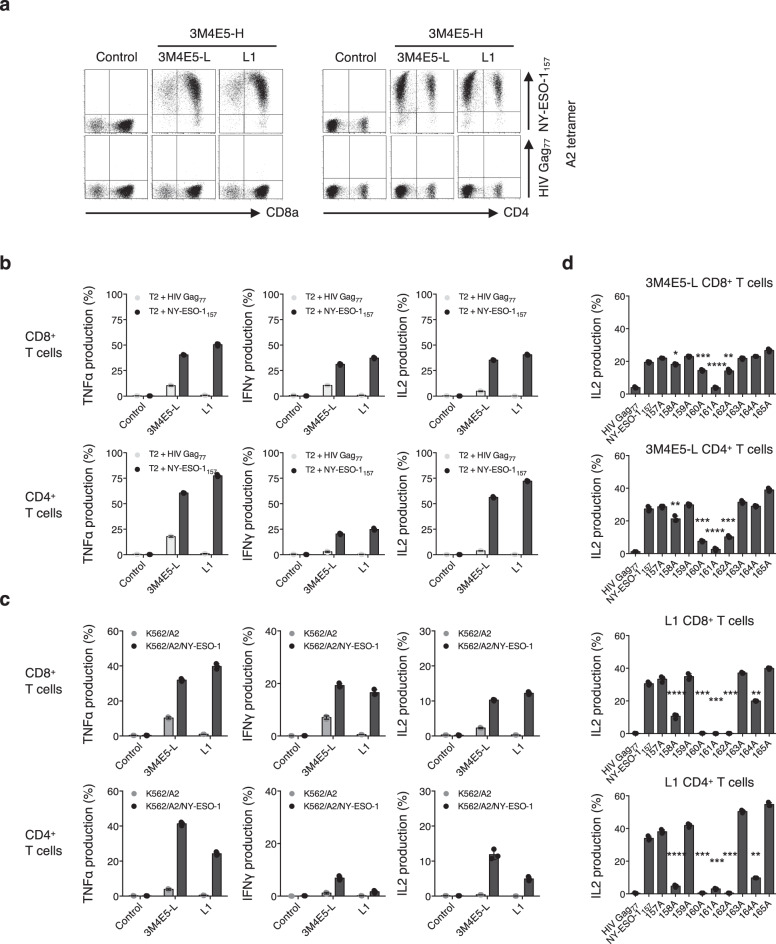
Sufficient antitumor reactivity and minimized cross-reactivity of fine-tuned scFv-expressing A2/NY-ESO-1_157_ CAR-T cells. **a** Clone L1, or the original 3M4E5-L second generation (CD28ζ) CAR was transduced into peripheral blood T cells. Control T cells and these transfectants were stained with 20 μg/mL A2/NY-ESO-1_157_ tetramer or A2/HIV Gag_77_ tetramer. Representative dot plots of both CD8^+^ T cells and CD4^+^ T cells are shown. **b**, **c** CAR-T cells generated as above were incubated with the indicated peptide-pulsed T2 cells (**b**), K562/A2 cells, or K562/A2/NY-ESO-1 cells (**c**), and their cytokine production was measured by intracellular cytokine assays. The experiments were performed in triplicate, and error bars depict the SD. **d** L1 CAR or original 3M4E5-L CAR CD8^+^ T cells and CD4^+^ T cells were incubated with T2 cells pulsed with 9 different alanine-substituted peptides at 10 μg/mL. IL2 production (%) was measured by intracellular cytokine assays. Each response to alanine-substituted peptides was compared with the response to the original NY-ESO-1_157_ peptide. The experiments were conducted in triplicate, and error bars depict the SD. Welch’s *t* test (two-sided) was performed for comparison. *p < 0.05; **p < 0.01; ***p < 0.001; ****p < 0.0001. Note that control T cells were generated by transduction of the *ΔNGFR* gene into human peripheral blood T cells. Gene-modified T cells with similar transduction efficiency were prepared, and then ΔNGFR-positive cells were gated and analyzed (**a**–**d**).

Next, we examined target-specific reactivity of human peripheral blood T cells redirected with newly isolated A2/NY-ESO-1_157_ scFv-encoding CAR. Clone L1, which showed sufficient structural and functional avidities for A2/NY-ESO-1_157_ when paired with 3M4E5-H, was selected. Both L1 and the original 3M4E5-LH CAR-transduced CD8^+^ and CD4^+^ T cells recognized A2/NY-ESO-1_157_ (Fig. 3a, b). Furthermore, we prepared K562/A2 cells and K562/A2/NY-ESO-1 cells, which were transduced with the HLA-A*02:01 gene with or without the *NY-ESO-1* gene, and used as target cells. Both L1 CAR-T cells and 3M4E5-L CAR-T cells recognized K562/A2/NY-ESO-1 cells. Importantly, L1 CAR-T cells did not show any reactivity with K562/A2 cells (Fig. 3c). These results suggest that cross-reactivity to HLA-A*02:01/peptide complexes differed between L1 CAR-T cells and 3M4E5-L CAR-T cells even though the same VH (3M4E5-H) was employed for their scFvs.

We then investigated in more detail the potential cross-reactivity of L1 CAR-T cells with peptides homologous to NY-ESO-1_157_ associated with an HLA-A*02:01 allele. Alanine scanning analysis was performed using 9 peptides in which each amino acid of NY-ESO-1_157_ peptide was substituted with alanine. L1 CAR-T cells had less cross-reactivity than the original 3M4E5-L CAR-T cells (Fig. 3d). Since the second, fourth, fifth, sixth, and eighth amino acids appeared to be important for recognition by L1 CAR-T cells, we searched for homologous peptides containing the xLxMWIxQx sequence. One cross-reactive candidate peptide, lysophospholipase-like protein 1 (LYPLAL1)-derived peptide (LYPLAL1_36-44_), was identified. L1 CAR-T cells recognized LYPLAL1_36_ peptide pulsed onto T2 cells, but did not recognize K562/A2/LYPLAL1 cells (Supplementary Fig. 4). These results suggest that target-specific recognition of CAR-T cells can be finely tuned by modifying a variable region in an scFv.

### Successful identification of new scFvs from CD19-specific CAR-library T cells

**Fig. 4:**
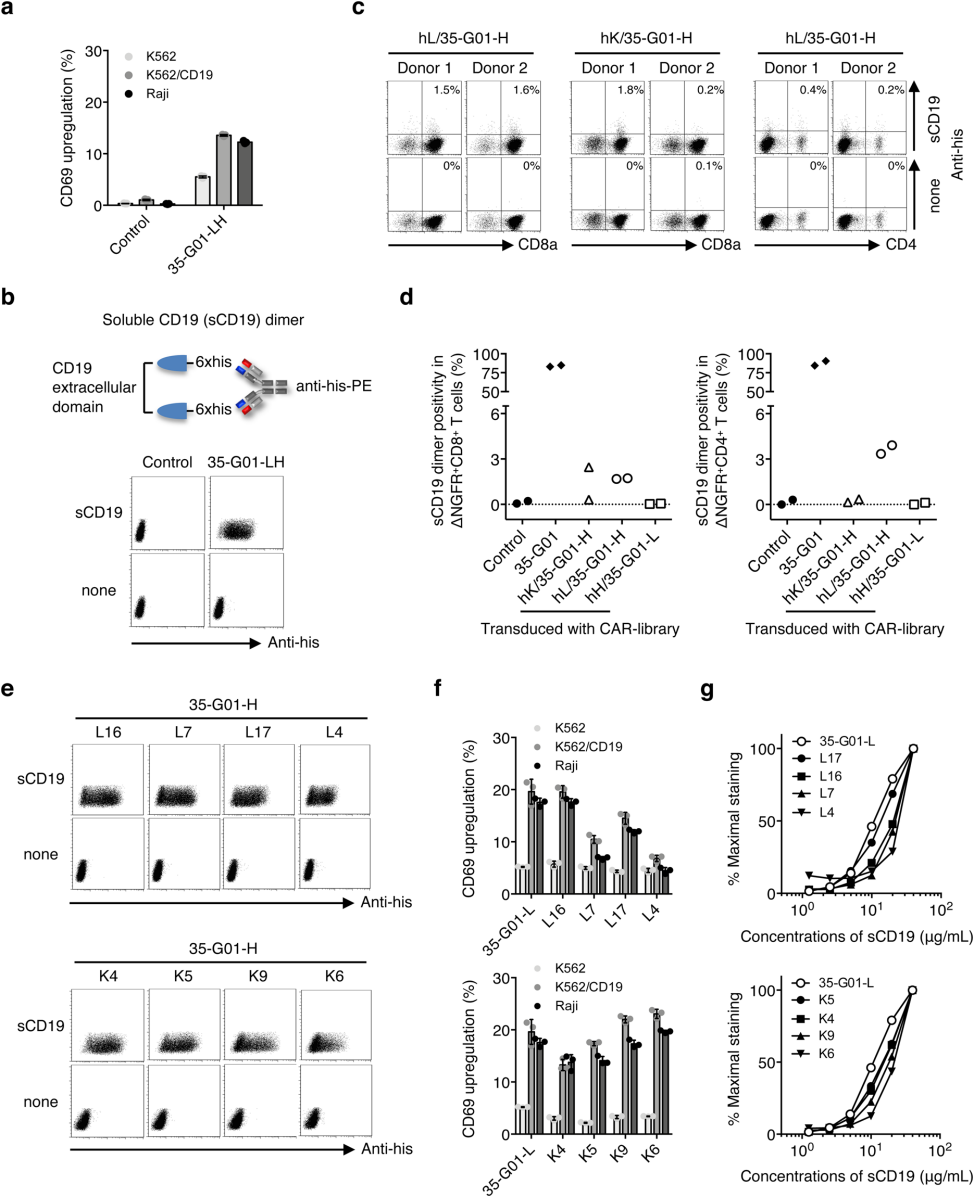
Target reactivity of CAR-T cells expressing new scFvs isolated from CD19 CAR-library T cells. **a** CD69 upregulation of Jurkat 76/35-G01-LH CAR cells and control Jurkat 76 cells in response to K562, K562/CD19, and Raji cells was measured by flow cytometry. The experiments were performed in triplicate, and error bars show the SD. **b** A construct of the soluble CD19 (sCD19) dimer is shown (top). Jurkat 76/CAR transfectants and control Jurkat 76 cells were stained with 40 μg/mL sCD19 dimer or PE-anti-his mAb alone (bottom). **c** CD19-specific scFv libraries possessing each human kappa chain (hK), lambda chain (hL) library derived from two different donors along with 35-G01-H, indicated as hL/35-G01-H and hK/35-G01-H, were individually generated. A second-generation CAR library encoding each CD19-specific scFv library together with CD28ζ was transduced into peripheral blood T cells. CD19 CAR-library T cells similarly stimulated three times were stained with 40 μg/mL sCD19 dimer or PE-anti-his mAb alone. Representative stainings of CD19 CAR-library CD8^+^ T cells (hL/35-G01-H and hK/35-G01-H) derived from donors 1 and 2, and CD4^+^ T cells (hL/35-G01-H) derived from the same donors are depicted. Each percentage of sCD19 dimer-positive cells among CAR-library T cells is shown. **d** sCD19 dimer positivity in CAR-library (hK/35-G01-H, hL/35-G01-H, and hH/35-G01-L) CD8^+^ T cells and CD4^+^ T cells is summarized. Each dot represents each library source derived from the different donors. The original 35-G01 CAR-T cells and control T cells were prepared as a positive and a negative control, respectively. **e** Newly isolated CD19 CARs were individually reconstituted in Jurkat 76 cells. Jurkat 76/CAR transfectants expressing each different VL (L16, L7, L17, L4, K4, K5, K9, and K6) chain paired with 35-G01-H were stained with 40 μg/mL sCD19 dimer or PE-anti-his mAb alone. L and K indicate lambda and kappa chain, respectively. **f** Jurkat 76/CAR transfectants were incubated with the indicated target cells, and their CD69 upregulation was measured by flow cytometry. The experiments were performed in triplicate, and error bars show the SD. **g** Jurkat 76/CAR transfectants were stained with graded concentrations of sCD19 dimer. Percentage maximal staining was calculated relative to the staining pattern of each transfectant with 40 μg/mL sCD19 dimer as 100%.

To demonstrate further advantages, we applied this scFv generation system to identify optimal scFvs for CD19-specific CAR-T cells which are clinically used for treatment of hematological malignancies. A variable region library (hL, hK, and hH) of two donors was linked with the VH or VL of CD19-specific antibody (35-G01-H or 35-G01-L), and integrated into the second generation (CD28ζ) CAR construct^39^ (Fig. 1a). A CAR-library derived from each donor was individually transduced into peripheral blood T cells to generate CD19-specific CAR-library T cells (Fig. 1b, c). The original 35-G01-LH CAR-transduced Jurkat 76 cells recognized both Raji cells and K562/CD19 cells that express CD19 (Fig. 4a; Supplementary Fig. 5a). Since the transfectants were successfully stained with a soluble CD19 (sCD19) dimer possessing two extracellular domains of the CD19 molecule (Fig. 4b), we utilized the sCD19 dimer to detect a CD19-specific population among CAR-library T cells. Soluble CD19 dimer-positive cells among CD19 CAR-library T cells were rare prior to stimulation (Supplementary Fig. 5b). After three stimulation of the CAR-library T cells with CD19^+^ APCs, CD19-specific CAR-library (hL/35-G01-H, hK/35-G01-H) CD8^+^ and CD4^+^ T cells were successfully detected with the sCD19 dimer (Fig. 4c, d).

Then, we isolated the sCD19 dimer-positive cells and determined the primary structures of the new scFvs (Fig. 1d). The variable regions and amino acid sequences of CDR3 are shown (Supplementary Table 2). Twelve different VLs were identified, and these new CARs were individually reconstituted in Jurkat 76 cells. Jurkat 76/CAR transfectants recognized CD19 with different degrees (Fig. 4e, f; Supplementary Fig. 5c, d). Structural avidities of Jurkat 76/CAR transfectants were measured using graded concentrations of sCD19 dimer, and showed differences between the transfectants (Fig. 4g; Supplementary Fig. 5e).

### Sufficient cytotoxicity, proliferation capacity, and survival advantage of scFv-optimized CD19 CAR-T cells

**Fig. 5:**
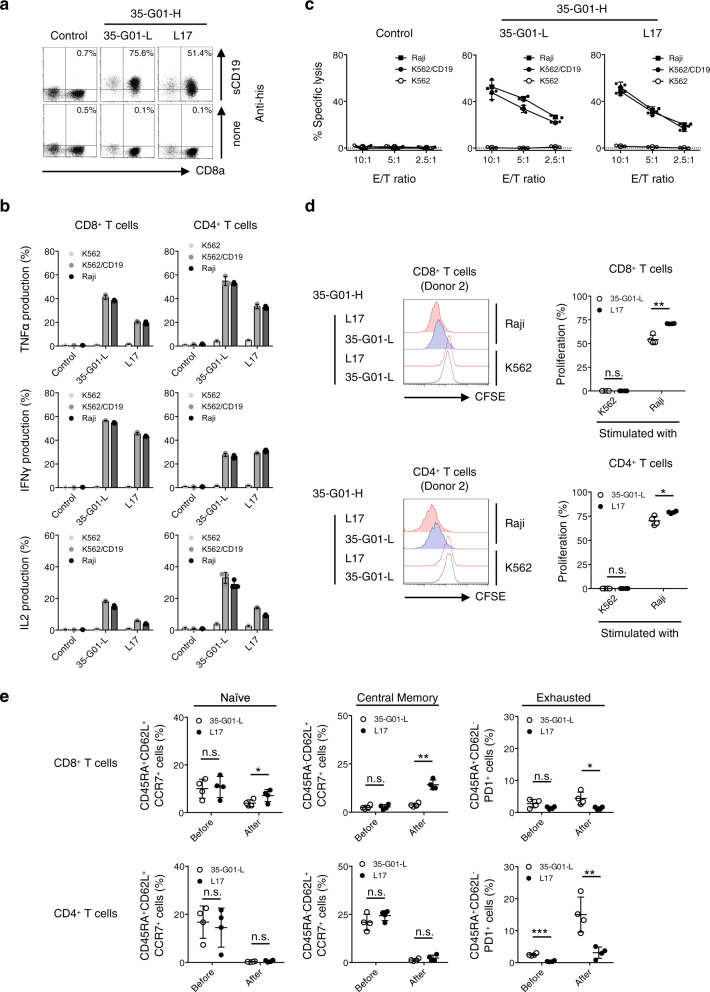
Antitumor reactivity, proliferation capacity, and durable phenotypes of scFv-optimized CD19 CAR-T cells. **a**, **b** Clone L17, or the original 35-G01-L CD19-specific second generation (CD28ζ) CAR was transduced into peripheral blood T cells, which were then stained with 40 μg/mL sCD19 dimer or PE-anti-his mAb alone, and representative dot plots are shown. Each percentage of sCD19 dimer-positive cells in ΔNGFR^+^ cells is displayed (**a**). L17, and 35-G01-L CD19 CAR-T cells were incubated with the indicated target cells, and their cytokine production was measured by intracellular cytokine assays. The experiments were performed in triplicate, and error bars show the SD (**b**). Control T cells were prepared by transduction of the *ΔNGFR* gene into human peripheral blood T cells. Gene-modified T cells with similar transduction efficiency were obtained, and then ΔNGFR-positive cells were gated and analyzed. **c** The cytotoxicity of established and isolated CD19 CAR-T cells against target cells was examined by ^51^Cr-release assays. The experiments were performed in triplicate, and error bars depict the SD. **d**, **e** L17, and 35-G01-L CAR-T cells established from four different donors were isolated, labeled with carboxyfluorescein diacetate succinimidyl ester (CFSE), and stimulated with irradiated Raji cells at an E/T ratio of 1:1. After 3 days of incubation, the mean fluorescence intensity (MFI) of CFSE-labeled CAR CD8^+^ T cells and CD4^+^ T cells was examined. The MFI values for each of the donor-derived CFSE-labeled CAR-T cells in the presence of K562 cells instead of Raji cells were utilized as a control. Histograms for CFSE-CAR CD8^+^ T cells and CD4^+^ T cells (donor 2) stimulated with Raji cells (filled lines), or K562 cells (dotted lines), are shown. Red represents the histograms of L17 CAR-T cells, and blue those of 35-G01-L CAR-T cells (left). The percentage of proliferation was calculated as: (control MFI−experimental MFI)/(control MFI) × 100 (%), and is summarized (right) (**d**). CD45RA^+^CD62L^+^CCR7^+^, CD45RA^-^CD62L^+^CCR7^+^, and CD45RA^+^CD62L^-^PD1^+^ populations among individual CAR CD8^+^ T cells and CD4^+^ T cells before and after stimulation with Raji cells are also summarized (**e**). Each dot depicts the percentage of the indicated population among CAR-T cells generated from each donor. Differences between L17 and 35-G01-L CAR-T cells were statistically assessed by paired *t* test (two-sided). *p < 0.05; **p < 0.01; ***p < 0.001; n.s., not significant.

Next, a representative clone, L17 and original 35-G01-L pairing with 35-G01-H in an scFv of second generation (CD28ζ) CAR were individually transduced into human peripheral blood T cells. Then, we examined whether the newly optimized scFv-expressing CAR-T cells exerted sufficient antitumor functions. Both L17 CAR-T cells and original 35-G01L CAR-T cells recognized CD19, and produced cytokines in response to Raji cells as well as K562/CD19 cells but not K562 cells (Fig. 5a, b). L17-expressing CAR-T cells produced lower amounts of cytokines but showed similar cytotoxicity against CD19^+^ tumor cells when compared with the original 35-G01-L CAR-T cells (Fig. 5b, c). Importantly, after stimulation of the CAR-T cells with CD19^+^ Raji cells, the L17 CAR-transduced CD8^+^ T cells and CD4^+^ T cells displayed superior proliferation capacity (Fig. 5d), and the frequencies of CD45RA^+^CD62L^+^CCR7^+^ (naïve) and CD45RA^-^CD62L^+^CCR7^+^ (central memory) CD8^+^ CAR-T cells were increased without any increase in CD45RA^+^CD62L^-^PD1^+^ exhausted CAR-T cells relative to 35-G01-L CAR-T cells (Fig. 5e; Supplementary Fig. 6). Furthermore, after two stimulations, L17 CAR-T cells proliferated, and retained their killing activity for CD19^+^ tumor cells but with less potential for cytokine production in comparison to similarly stimulated 35-G01-L CAR-T cells (Supplementary Fig. 7). Collectively, scFv-optimized CAR-T cells showed sufficient antitumor cytotoxicity, superior proliferation capacity, and survival advantage. Interestingly, the ability to produce large amounts of cytokines does not necessarily predict these superior antitumor functions of CAR-T cells.

### Enhanced in vivo antitumor functions induced by scFv-optimized CD19 CAR-T cells

**Fig. 6:**
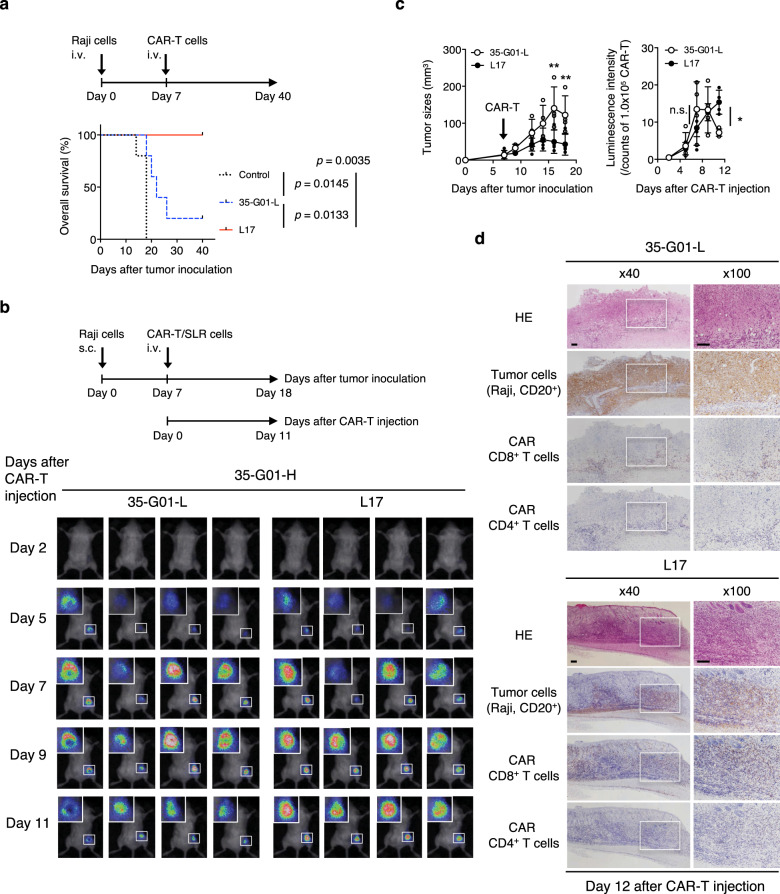
Superior in vivo antitumor functions mediated by scFv-optimized CD19 CAR-T cells. **a** Five hundred thousand Raji cells were intravenously injected into irradiated NOD/Shi-scid IL2rgamma(null) mice. After engraftment of the Raji cells, 2.0 × 10^6^ L17, 35-G01-L CAR-T cells, or control T cells generated from the same donor were intravenously injected on Day 7. Overall survival of the mice (*n* = 5 mice per group) is depicted in the form of Kaplan–Meier curves, and survival was compared among the groups. Log-rank (Mantel–Cox) test was performed, and *p* values are also shown. **b** One million Raji cells were subcutaneously inoculated into irradiated NOD/Shi-scid IL2rgamma(null) mice, then 2.0 × 10^6^ L17 or 35-G01-L CAR-T cells, which can express SLR, were intravenously injected on Day 7. Photon images of mice obtained after CAR-T-cell injection are shown (*n* = 4 mice per group). The small white rectangles are magnified and shown in the upper left corner for each mouse. **c** Tumor sizes (mm^3^) (left) and photon counts normalized by each photon count obtained from 1.0 × 10^5^ L17 or 35-G01-L CAR-T cells (right) are shown. The arrow inside the left-hand graph indicates the time at which CAR-T cells were injected. Error bars show the SD. Two-way ANOVA with Sidak’s correction was conducted for multiple comparisons. *p < 0.05; **p < 0.01; n.s., not significant. **d** Mice shown in (**b**) were sacrificed on Day 12 after CAR-T injection, and tumor tissues isolated from the right flanks were assessed histologically. Representative immunostained sections (hematoxylin and eosin (HE); CD20 for tumor cells; CD8 and CD4 for CAR-T cells) obtained from tissues in a representative mouse from each group are shown. The white rectangles (left, ×40) are displayed as magnified images (right, ×100). The scale bars in the pictures represent 100 μm.

To confirm the antitumor effects in vivo, we then performed side-by-side experiments using established CD19 CAR-T cells or control T cells that were intravenously injected into tumor-bearing NOD/Shi-scid IL2rgamma(null) mice on Day 5 of tumor-cell injection. Both L17 and 35-G01-L CAR-T cells, but not control T cells, showed antitumor effects and prolonged survivals (Supplementary Fig. 8). Importantly, mice bearing advanced tumors treated with L17 CAR-T cells on Day 7 displayed extended survival as compared with mice similarly treated with the original 35-G01-L CAR-T cells (Fig. 6a). To assess whether scFv-optimized CAR-T cells show in vivo antitumor effects even against mass-forming tumors, we prepared NOD/Shi-scid IL2rgamma(null) mice subcutaneously inoculated with CD19^+^ Raji cells. After intravenous injection of luciferase-transduced CAR-T cells, L17 CAR-T cells, but not 35-G01-L CAR-T cells, suppressed tumor growth and showed greater infiltration of the tumor site on Day 11 (Fig. 6b, c). Histological analyses performed on Day 12 also revealed that residual tumor cells were surrounded by a small number of 35-G01-L CD8^+^ CAR-T cells, while L17 CAR-T cells appeared to diffusely infiltrate into the diminished tumors (Fig. 6d). Therefore, an optimized scFv is able to maximize the in vivo antitumor functions of CAR-T cells.

## Discussion

In the present study, we have developed the T-cell-based scFv generation system to establish the ideal CAR-T-cell immunotherapy and revealed new findings as follows. First, unlike the conventional methods, this system is applicable to concentrate scFvs optimal for CAR-T cells by antigen-specific stimulation of a CAR-library expressing human peripheral blood T cells. Second, target-specific recognition of CAR-T cells can be finely tuned by selecting variable regions in their scFvs. Third, scFv-optimized CAR-T cells show sufficient antitumor cytotoxicity, superior proliferation capacity, and durable phenotypes, resulting in enhanced in vivo antitumor effects against advanced and mass-forming tumors even those expressing the same spacer, transmembrane, and intracellular domains.

Since L1 CAR-T cells possessing the new variable region L1 with 3M4E5-H responded to a large amount of LYPLAL_36_ peptide pulsed onto T2 cells, L1 CAR-T cells have minimized but potential cross-reactivity. However, L1 CAR-T cells recognized K562/A2/NY-ESO-1 cells but not K562/A2/LYPLAL cells, probably due to the low capacity of LYPLAL_36_ peptide to bind to the HLA-A*02:01 molecule (Supplementary Fig. 4). These results suggest the safety and sufficient A2/NY-ESO-1_157_ reactivity of L1 CAR-T cells when compared with the original 3M4E5-L CAR-T cells. On the other hand, L52 CAR-T cells recognized HLA-A*02:01/peptide complexes when pairing with the 3M4E5-H. Based on the crystal structure analyses of scFvs specific for MHC class I/peptide complexes, different types of scFv footprints were reported. That is, most of scFvs recognizes both a MHC class I molecule and a target peptide^40^, in contrast, some other scFvs appear to recognize mainly the alpha-helix region of an MHC class I^40–42^. These scFvs whose structures are tilted to an alpha 1 or alpha 2 helix might show less dependency on target peptides presented by an MHC class I. A previous report showed that the scFv, clone 3M4E5 employed in this study, recognizes NY-ESO-1_157_ peptide as well as the HLA-A*02:01 molecule on the basis of its footprint^32^. Therefore, our results suggest that L1 can force the scFv structure to shift towards the presented peptide more closely, whereas L52 may tilt the scFv towards an alpha 1 or 2 helix of the HLA-A*02:01 molecule while sharing the same VH, 3M4E5-H.

A recent study has demonstrated that reactivity of CAR-T cells can be modulated by changing amino acids in CDR3 region of VH^43^. In contrast, our system allows us to adjust a variable region including CDR1 and CDR2 as well as CDR3 with different length and amino acid sequences. This suggests that it may not be needed to manipulate the length of linker and spacer sequences in extracellular domains of a CAR construct in our system. In other words, our scFv generation system using CAR-library T cells would comprehensively manipulate the interaction between CARs expressed by T cells and target antigens presented by tumor cells, resulting in selection of scFvs with fine-tuned immunological synapses^13,44^. Although in this study we employed the second generation CD28ζ CAR, it would be possible to generate new scFvs optimal for other types of CARs possessing different intracellular domains^15–21^. In addition, proteins specifically glycosylated in tumor cells have been previously identified, and CAR-T-cell therapy targeting such glycosylated tumor antigens has been developed^45^. This system may also be applicable for optimizing scFvs expressed by CAR-T cells targeting tumor-specific glycosylation. Another advantage of our system is that this would be exploited to easily generate new scFvs which possess human immunoglobulin-derived variable regions. Therefore, it may be possible to convert murine scFvs into completely human immunoglobulin-derived scFvs to avoid xenoreactive adverse events^23^.

Our study has emphasized that an scFv plays important roles in inducing superior CAR-T-cell functions. We have also shown that cytokine production capacity and superior in vivo antitumor functions are not always necessarily correlated. Our results suggest that the sufficient antitumor cytotoxicity, superior proliferation capacity, and durable phenotypes of scFv-optimized CAR-T cells are likely to account for their accumulation at the tumor site and suppression of tumor growth in vivo. It should be noted that our in vitro scFv generation system facilitates the enrichment of scFv-optimized CAR-T cells, but not suboptimal populations since scFv-optimized CAR-T cells have a survival advantage and can proliferate while retaining their antitumor cytotoxicity after repeated stimulations (Supplementary Fig. 9). In addition, treatment of patients using scFv-optimized CAR-T cells with lower capacity for cytokine production, such as L17 CD19 CAR-T cells, may reduce the risk of severe cytokine-release syndrome^46,47^. Therefore, this in vitro system can be useful for identifying new scFvs with the potential for effective and safe CAR-T-cell immunotherapy. However, further in vivo studies will be needed to assess not only the efficacy and toxicity but also the long-term persistence of scFv-optimized CAR-T cells. In addition, on-target and/or off-target adverse events induced by newly generated CAR-T cells in the human body still remain unknown. Clinical trials using CAR-T cells expressing an scFv optimized by exploiting this system will clarify this point.

In conclusion, we have demonstrated that an optimization of scFv for CAR-T cells is needed to maximize the in vivo antitumor functions of CAR-T cells. Our T-cell-based scFv generation system may be of value for optimal tuning of an immunological synapse formed by an scFv expressed by CAR-T cells and a target antigen expressed by a tumor cell, thus representing an ideal form of CAR-T-cell immunotherapy against advanced malignancies.

## Methods

### Cells

K562 cells, which lack expression of endogenous HLA and CD19, were purchased from the American Type Culture Collection (ATCC) and cultured in RPMI1640 supplemented with 10% FCS. T2 cells, which endogenously express HLA-A*02:01 as one of the HLA alleles, were cultured in RPMI1640 supplemented with 10% FCS. Raji cells, which endogenously express CD19 and CD20, were cultured in RPMI 1640 supplemented with 10% FCS. Plat-A cells (kindly provided by Dr. Toshio Kitamura, Institute of Medical Sciences, University of Tokyo) were maintained in DMEM supplemented with 10% FCS, 1 μg/mL puromycin, and 10 μg/mL blasticidin^48^. PG13 cells were cultured in DMEM supplemented with 10% FCS. Jurkat 76 cells, which lack endogenous TCR and CD3 expression (a generous gift from Dr. Mirjam Heemskerk, Leiden University), were cultured with RPMI1640 supplemented with 10% FCS^38^. Human peripheral blood mononuclear cells (PBMCs) were isolated from healthy volunteers using Ficoll-Paque (GE Healthcare) and stored until use. All of the experiments using human samples were approved by the Certified Review Board of Ehime University. All experiments were performed in accordance with the relevant guidelines and regulations. All of the donors provided written informed consent.

### cDNAs

The antigen-specific CAR was generated as described previously^33^. Briefly, variable and J regions derived from the immunoglobulin light and heavy chains of an HLA-A*02:01-restricted NY-ESO-1_157-165_ (SLLMWITQC) (A2/NY-ESO-1_157_)-specific monoclonal antibody (mAb) (clone 3M4E5) or a human CD19-specific mAb (clone 35-G01) were linked with a linker sequence (GSTSGSGKPGSGEGSTKG) to generate the scFv^31–33,39^. A human mesothelin-specific scFv (clone m912) was employed as an irrelevant scFv^49^. Antigen-specific second-generation CARs encoding each scFv followed by a transmembrane region of CD28 and an intracellular domain of the CD3ζ chain (CD28ζ) were synthesized using GeneArt (Thermo Fisher Scientific). To detect CAR-transduced T cells, a *CAR* gene was fused with a truncated nerve growth factor receptor (*ΔNGFR*) gene via a furin cleavage site, an SGSG spacer sequence, and a codon-optimized P2A sequence^33^. Genes of interest which are forced to be expressed intracellularly were similarly fused with the *ΔNGFR* gene. To assess in vivo accumulation of CAR-T cells, a *CAR* gene was similarly fused with a *luciferase* (*SLR: stable luciferase red*) gene and the *ΔNGFR* gene. Antigen-specific CAR-T cells among CAR-library T cells were collected by flow cytometry. To isolate new antigen-specific scFv genes after cDNA generation, PCR was performed using a set of primers, 5′ pMX (ATCCCAGTGTGGTGGTACGGG) and 3′ CD28ζ (GGGTACATCACTTCGATTGC). ScFv amplicons were individually cloned again into the same second generation (CD28ζ) CAR construct with the pMX backbone vector. Their nucleic acid sequences were determined using an ABI 3500 (Applied Biosystems).

### Antigen-specific scFv library

To generate a variable region library derived from the immunoglobulin light/heavy chain, human peripheral blood B cells were purified using anti-CD19 microbeads (Miltenyi Biotec). Around 3.0 × 10^6^ CD19^+^ B cells were collected and their RNAs were isolated, from which cDNA was generated using a SMARTer RACE cDNA amplification kit (Takara Bio). The variable and J regions derived from the immunoglobulin heavy chain were amplified using a mixture of a 5′-RACE primer, 3′ IGHG (TGAGTTCCACGACACCGTCAC), and 3′ IGHM (TGATGGAGTCGGGAAGGAAGTC) primers specific for each CH1 region of IgG and IgM, respectively. The second-round semi-nested PCR was performed using the first PCR product as a template, a modified 5′-RACE primer (GTGTGGTGGTACGGGAATTCAAGCAGTGGTATCAACGCAGAGT), and each 3′ IGHJ1 (CTTGCCAGAGCCGCTTGTAGAGCCTGAGGAGACGGTGACCAGGG), 3′ IGHJ2 (CTTGCCAGAGCCGCTTGTAGAGCCTGAAGAGACGGTGACCATTGTC), or 3′ IGHJ3 (CTTGCCAGAGCCGCTTGTAGAGCCTGAGGAGACGGTGACCGTGGTC) primer, respectively, specific for each J region of the heavy chain. Variable and J regions derived from immunoglobulin light chain genes were amplified using the same methods, except for the 3′ primer, which was IGKC (AGGGTCAGAGGCCAAAGGATGG) or IGLC (CTTGGAGCTCCTCAGAGGAG) specific for each CL region of kappa or lambda for the first PCR, IGKJ1 (CTTGCCAGAGCCGCTTGTAGAGCCTTTGATTTCCACCTTGGTCCC), IGKJ2 (CTTGCCAGAGCCGCTTGTAGAGCCTTTGATCTCCAGCTTGGTCCC), IGKJ3 (CTTGCCAGAGCCGCTTGTAGAGCCTTTGATATCCACTTTGGTCCC), IGKJ4 (CTTGCCAGAGCCGCTTGTAGAGCCTTTAATCTCCAGTCGTGTCCC), IGLJ1 (CTTGCCAGAGCCGCTTGTAGAGCCTAGGACGGTGACCTTGGTCCC), IGLJ2 (CTTGCCAGAGCCGCTTGTAGAGCCTAGGACGGTCAGCTTGGTCCC), IGLJ4 (CTTGCCAGAGCCGCTTGTAGAGCCTAAAATGATCAGCTGGGTTCC), IGLJ5 (CTTGCCAGAGCCGCTTGTAGAGCCTAGGACGGTCAGCTCGGTCCC), or IGLJ7 (CTTGCCAGAGCCGCTTGTAGAGCCGAGGACGGTCAGCTGGGTGCC) specific for each J region of the light chain for the second PCR. The mixture of amplicons derived from the variable and J regions of the heavy chain or light chain located at the N-terminus was fused with the A2/NY-ESO-1_157_-specific variable region (3M4E5-L or 3M4E5-H) or the CD19-specific variable region (35-G01-L or 35-G01-H) via a linker sequence as described above by performing overlap extension PCR, followed by standard PCR using a set of primers, a secondary modified 5′-RACE primer (ATCCCAGTGTGGTGGTACGGGAATTCAAG) and 3′ CD28ζ (GGGTACATCACTTCGATTGC) to generate antigen-specific scFv libraries. An scFv library was directly subcloned into the second generation (CD28ζ) CAR construct with the pMX backbone vector treated with EcoRI/NotI enzymes using NEBuilder HiFi DNA Assembly Master Mix (New England Biolabs). Ten-beta high efficiency competent *E. coli* (New England Biolabs) was transformed, followed by harvesting of all the colonies obtained to generate a pMX-based CAR library. The heterogeneity of scFv regions in the CAR library was confirmed by Sanger sequencing (Applied Biosystems). The pMX-based CAR library was used for transduction into human peripheral blood T cells.

### Transfectants

K562 cells were retrovirally transduced with HLA-A*02:01 or CD19 together with costimulatory molecules, CD80 and CD83, to establish APCs for expansion of antigen-specific CAR-transduced T cells^35^. In some experiments, K562 cells were transduced with the HLA-A*02:01 gene to establish K562/A*02:01 (K562/A2), and K562/A2 cells were transduced with the *NY-ESO-1* gene or *LYPLAL1* gene to establish K562/A2/NY-ESO-1 cells and K562/A2/LYPLAL1 cells, respectively^33^. K562/CD19 cells, in which K562 cells were transduced with the *CD19* gene, were used as CD19^+^ target cells. Jurkat 76 cells were retrovirally transduced with newly isolated *CAR* genes or a control gene (*ΔNGFR* alone) to assess their target-specific reactivity. Raji cells were transduced with the SLR to establish Raji/SLR cells for in vivo bioluminescence imaging assays. Human peripheral blood T cells were stimulated with 100 IU/mL human IL-2 (Roche) and 50 ng/mL anti-human CD3ε mAb (clone OKT3) for 2 days before transduction. Then, T cells were cultured in RPMI1640 supplemented with 10% AB type human serum (Sigma Aldrich) and retrovirally transduced with *CAR* genes or a control gene (*ΔNGFR* alone) to establish gene-modified T cells^36,37^. Retronectin (Takara Bio) was utilized to transduce newly identified or original CD19-specific *CAR* genes into peripheral blood T cells. When the CAR library was transduced into peripheral blood T cells, a transduction efficiency of about 30% was achieved to theoretically express one CAR construct in one T cell^34^.

### Expansion of antigen-specific CAR-T cells

CAR-T cells were labeled with FITC-conjugated anti-human NGFR mAb (clone ME20.4) and isolated using anti-FITC microbeads (Miltenyi Biotec). Isolated CAR-library T cells were stimulated with the K562-based APCs established as above at an E/T ratio of 20:1^35–37^. To expand the A2/NY-ESO-1_157_-specific CAR-library T cells, A2-APCs were pulsed with 10 μg/mL NY-ESO-1_157_ peptide, irradiated, and the floating peptide was removed to stimulate T cells. To expand the CD19-specific CAR-library T cells, CD19-APCs were utilized without loading any peptides. These CAR-library T cells were cultured in RPMI1640 supplemented with 50 μg/mL gentamicin and 10% human AB serum in the presence of 10 IU/mL human IL-2 and 10 ng/mL human IL-15 (PeproTech). Following three stimulations, antigen-specific CAR-library T cells were used for analyses. In some experiments, newly identified CD19 CAR-T cells as well as the original CD19 CAR-T cells were stimulated with Raji cells (E/T 1:1) or CD19-APCs (E/T 10:1). After stimulation, the proliferation activity of the CD19 CAR-T cells was assessed by calculation of the fold increase.

### Flow cytometry

Antigen-specific CAR-library T cells were stained with PC5-anti-human CD8 mAb (clone B9.11), FITC-anti-human CD4 mAb (clone OKT4), and V450-anti-human NGFR mAb (clone C40-1457). APC-anti-human CD3 mAb (clone UCHT1) together with the other surface markers was utilized when confirming CD3 positivity. Biotinylated HLA-A2/NY-ESO-1_157_ monomer and HLA-A2/HIV Gag_77_ monomer (MBL) were multimerized using PE-conjugated streptavidin (Thermo Fisher Scientific) and utilized for staining of A2/NY-ESO-1_157_ CAR-T cells^33^. Soluble CD19 encodes the extracellular domain of CD19 followed by an SGSG linker sequence and a 6× histidine (his) tag at the C-terminus of the construct for detection and purification. Soluble CD19 dimer was generated by mixing sCD19 and PE-conjugated anti-his mAb (clone GG11-8F3.5.1) at a molar ratio of 2:1, then utilized for staining of CD19 CAR-T cells. A2/NY-ESO-1_157_ tetramer or sCD19 dimer-positive cells were isolated using FACSAria (Becton Dickinson). In some experiments, K562 transfectants were stained with PE-anti-HLA-A2 mAb (clone BB7.2) or PE-anti-human NGFR mAb (clone ME20.4). Raji cells and K562/CD19 cells were stained with PE-anti-human CD19 mAb (clone HIB19). To assess antigen-specific cytokine production by CAR-T cells in response to target cells, 3.0 × 10^5^ responder cells were incubated with 5.0 × 10^4^ target cells. Brefeldin A (BFA) was added after 2 h, and the cells were incubated for an additional 16 h to produce cytokines. Then, PE-anti-human TNFα mAb (clone MAb11), APC-anti-human IL2 mAb (clone MQ1-17H12), and PC7-anti-human IFNγ mAb (clone B27) together with the other surface markers described above were utilized. To assess the proliferation capacity and phenotype of the CAR-T cells, ΔNGFR^+^ CAR-T cells were collected and labeled with carboxyfluorescein diacetate succinimidyl ester (CFSE). Then, 2.0 × 10^5^ CFSE-labeled CAR-T cells were stimulated with irradiated Raji cells or K562 cells at an E/T ratio of 1:1. No cytokines were added. Three days later, CFSE-labeled CAR-T cells were stained with PE-anti-human PD1 mAb (clone EH12.2H7), APC-anti-human CD45RA mAb (clone HI100), PC7-anti-human CCR7 mAb (clone G043H7), BV421-anti-human CD62L mAb (clone DREG-56), and APC-Cy7-anti-human CD4 mAb (clone RPA-T4) in combination with PC5-anti-human CD8 mAb as described above. The CAR-transduced Jurkat 76 cells (Jurkat 76/CAR cells) were stained with A2/NY-ESO-1_157_ tetramer or sCD19 dimer along with V450 anti-human NGFR mAb. The antigen-specific reactivity of Jurkat 76/CAR cells for target cells was evaluated by measuring their CD69 upregulation using FITC-anti-human CD69 mAb (clone FN50). All samples were analyzed using a Gallios flow cytometer (Beckman Coulter) and FlowJo Version 7.6.5 software (TreeStar). ΔNGFR-positive cells were gated to assess CAR-T cells.

### Cytotoxicity assays

Standard cytotoxicity assays were performed as described previously^33^. Briefly, 5.0 × 10^3^ target cells were labeled with chromium-51 (^51^Cr) for 1.5 h at 37 °C. CAR-T cells were isolated and incubated with target cells at various E/T ratios for 6 h. The supernatants were then collected and their cpm counts were measured. The percentage of specific lysis was calculated as: (experimental release cpm–spontaneous release cpm)/(maximal release cpm−spontaneous release cpm) × 100 (%).

### In vivo murine experiments

All the murine experiments in this study were approved by the Ehime University Animal Care Committee. All experiments were performed in accordance with the relevant guidelines and regulations. Five-week-old NOD/Shi-scid IL2rgamma(null) female mice were purchased from In-Vivo Science Inc. and subjected to 1.5 Gy of irradiation. Then, 5.0 × 10^5^ Raji/SLR, or Raji cells were intravenously injected into the mice. After engraftment, 2.0 × 10^6^ isolated CAR-T cells, or control T cells with a similar CD4/CD8 ratio were intravenously injected into each mouse. Tumor sizes were measured by AEQUORIA-2D/8600 bioluminescence imaging assays (Hamamatsu Photonics). In some experiments, 1.0 × 10^6^ Raji cells were subcutaneously inoculated into the right flank of irradiated NOD/Shi-scid IL2rgamma(null) mice. Then, 2.0 × 10^6^ SLR-transduced and purified new or original CD19 CAR-T cells with a similar CD4/CD8 ratio were intravenously injected. The accumulation status of CAR-T cells in vivo was assessed by bioluminescence imaging assays as described above. The mice were sacrificed on Day 12, and their tumor tissues were assessed histologically.

### Immunohistochemistry

Subcutaneous tumor tissues obtained from the right flanks of mice were sectioned and stained as reported previously^50^. Briefly, sections were cut from formalin-fixed paraffin-embedded samples, deparaffinized, subjected to antigen retrieval, and incubated with anti-human CD20 mAb (clone L26), anti-human CD8 mAb (clone C8/144B), or anti-human CD4 mAb (clone 4B12), respectively. Treated sections were additionally stained with an EnVision+ Kit (horseradish peroxidase; mouse) (Agilent Technologies), and positive signals were obtained by adding diaminobenzidine tetrahydrochloride. The slides were counterstained with hematoxylin. No background staining was observed, as confirmed by side-by-side experiments using the isotype mAb.

### In silico analyses

The ScanProsite tool (http://prosite.expasy.org/scanprosite/) was utilized to search for human-derived peptide sequences homologous to NY-ESO-1_157_ peptide (SLLMWITQC) based on the results of alanine scanning assays. The binding affinity of peptides for HLA-A*02:01 was predicted by netMHC4.0 (http://www.cbs.dtu.dk/services/NetMHC/).

### Statistics and reproducibility

Statistical analyses were performed with GraphPad Prism 6.0h. Welch’s *t* test (two-sided) or paired *t* test (two-sided) was employed to examine whether two groups subject to parametric variation were significantly different for a given variable. Two-tailed Mann–Whitney test was performed to compare two different groups with nonparametric variation. Two-way ANOVA with Sidak’s correction was conducted for multiple comparisons. Murine survival was expressed in the form of Kaplan–Meier curves, and log-rank (Mantel–Cox) test was performed. Two-tailed nonparametric Spearman correlation test was performed to evaluate correlations between two independent variables. For each statistical analysis, a *p* value of <0.05 was considered significant. Statements of reproducibility including biologically independent sample sizes and replicates are shown in the figure legends.

### Reporting summary

Further information on research design is available in the Nature Research Reporting Summary linked to this article.

## Supplementary information

Supplementary Information

Description of Additional Supplementary Files

Supplementary Data 1

Reporting Summary

## Data Availability

Source data for graphs in the main figures are available in the Supplementary Data 1. All data supporting the conclusions of this study are included in the manuscript and its supplementary files, or are available from the corresponding author upon reasonable request.
