# Supplementary material for: A single-chain antibody generation system yielding CAR-T cells with superior antitumor function
Source: Commun Biol. 2021 Mar 2;4:273. doi: 10.1038/s42003-021-01791-1 (PMC7925539; doi:10.1038/s42003-021-01791-1)
Supplement: Supplementary file 1 — Supplementary Information [file 42003_2021_1791_MOESM1_ESM.pdf]

A single-chain antibody generation system yielding CAR-T cells with superior antitumor function

Toshiki Ochi[1,2], Masaki Maruta[1], Kazushi Tanimoto[1], Fumitake Kondo[1], Toshihiro Yamamoto[3], Mie Kurata[3,4], Hiroshi Fujiwara[1,5], Junya Masumoto[3,4], Katsuto Takenaka[1], Masaki Yasukawa[2,6]

[1]Department of Hematology, Clinical Immunology, and Infectious Diseases, Ehime University Graduate School of Medicine, Toon, Ehime 791-0295, Japan; [2]Division of Immune Regulation, Proteo-Science Center, Ehime University, Toon, Ehime 791-0295, Japan; [3]Department of Analytical Pathology, Ehime University Graduate School of Medicine, Ehime University, Toon, Ehime 791-0295, Japan; [4]Division of Pathology, Proteo-Science Center, Ehime University, Toon, Ehime 791-0295, Japan; [5]Department of Personalized Cancer Immunotherapy, Mie University Graduate School of Medicine, Tsu, Mie 514-8507, Japan; [6]Ehime Prefectural University of Health Sciences, Tobe, Ehime 791-2101, Japan.

Corresponding author

Toshiki Ochi, MD, PhD

Department of Hematology, Clinical Immunology and Infectious Diseases,

Ehime University Graduate School of Medicine

Shitsukawa, Toon, Ehime 791-0295, Japan

Phone: +81-89-960-5296

Fax: +81-89-960-5299

E-mail: ochi.toshiki.eg@ehime-u.ac.jp

Supplementary Figure 1

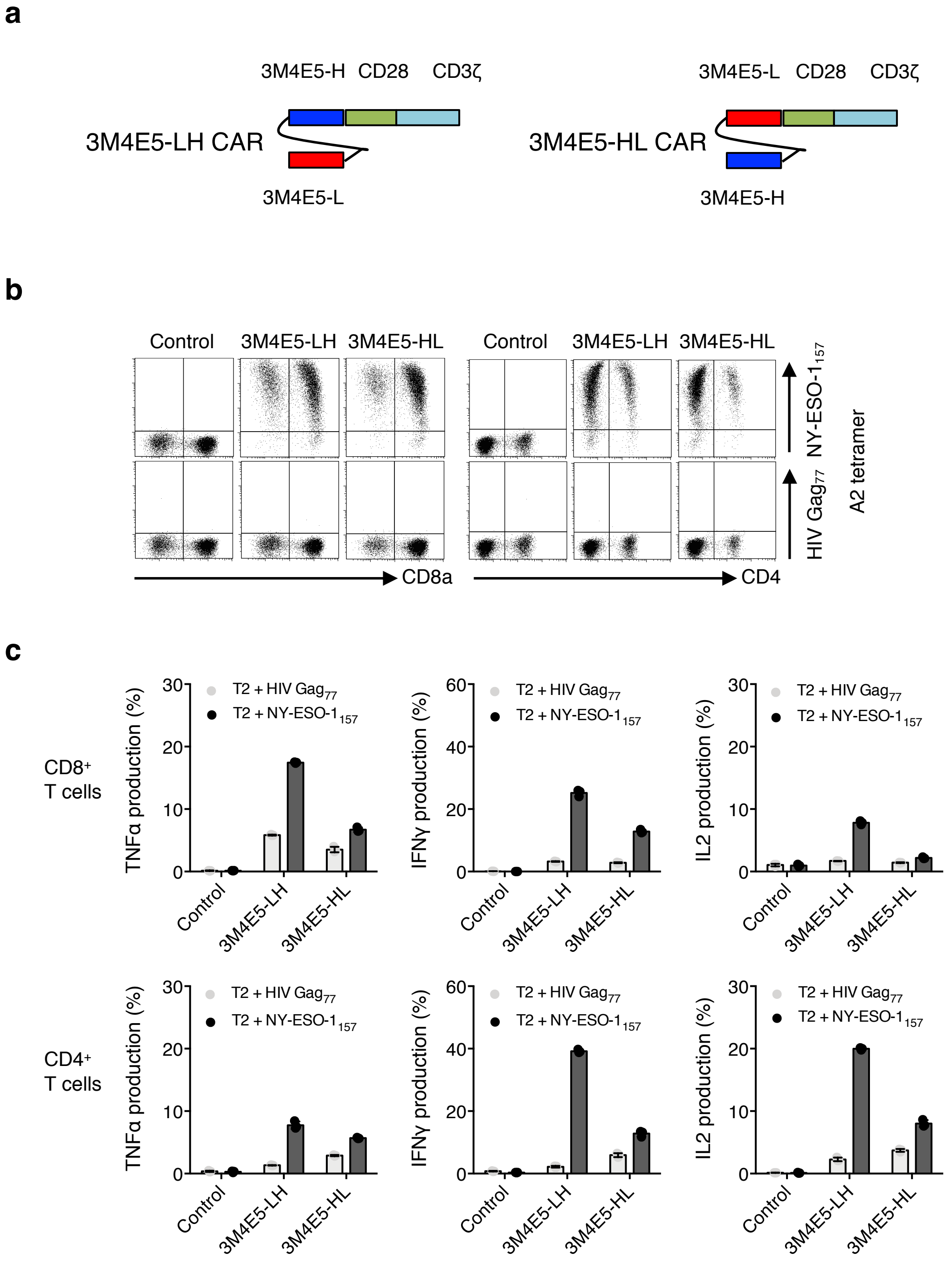

Supplementary Figure 1. 3M4E5 CAR-T cells recognized A2/NY-ESO-1_{157}.

a Second-generation CAR (clone 3M4E5) specific for A2/NY-ESO-1_{157}. An A2/NY-ESO-1_{157}-specific scFv (3M4E5-LH or 3M4E5-HL) was fused with the CD28 and CD3ζ chains. **b** A2/NY-ESO-1_{157}-specific CAR $CD8^+$ T cells and $CD4^+$ T cells were stained with 5 μg/mL A2/NY-ESO-1_{157} tetramer or A2/HIV Gag_{77} tetramer. **c** A2/NY-ESO-1_{157} CAR-T cells were incubated with T2 cells loaded with 10 μg/mL NY-ESO-1_{157} peptide or HIV Gag_{77} peptide, and their cytokine production was measured by intracellular cytokine assays. The experiments were performed in triplicate, and error bars demonstrate the SD.

Supplementary Figure 2

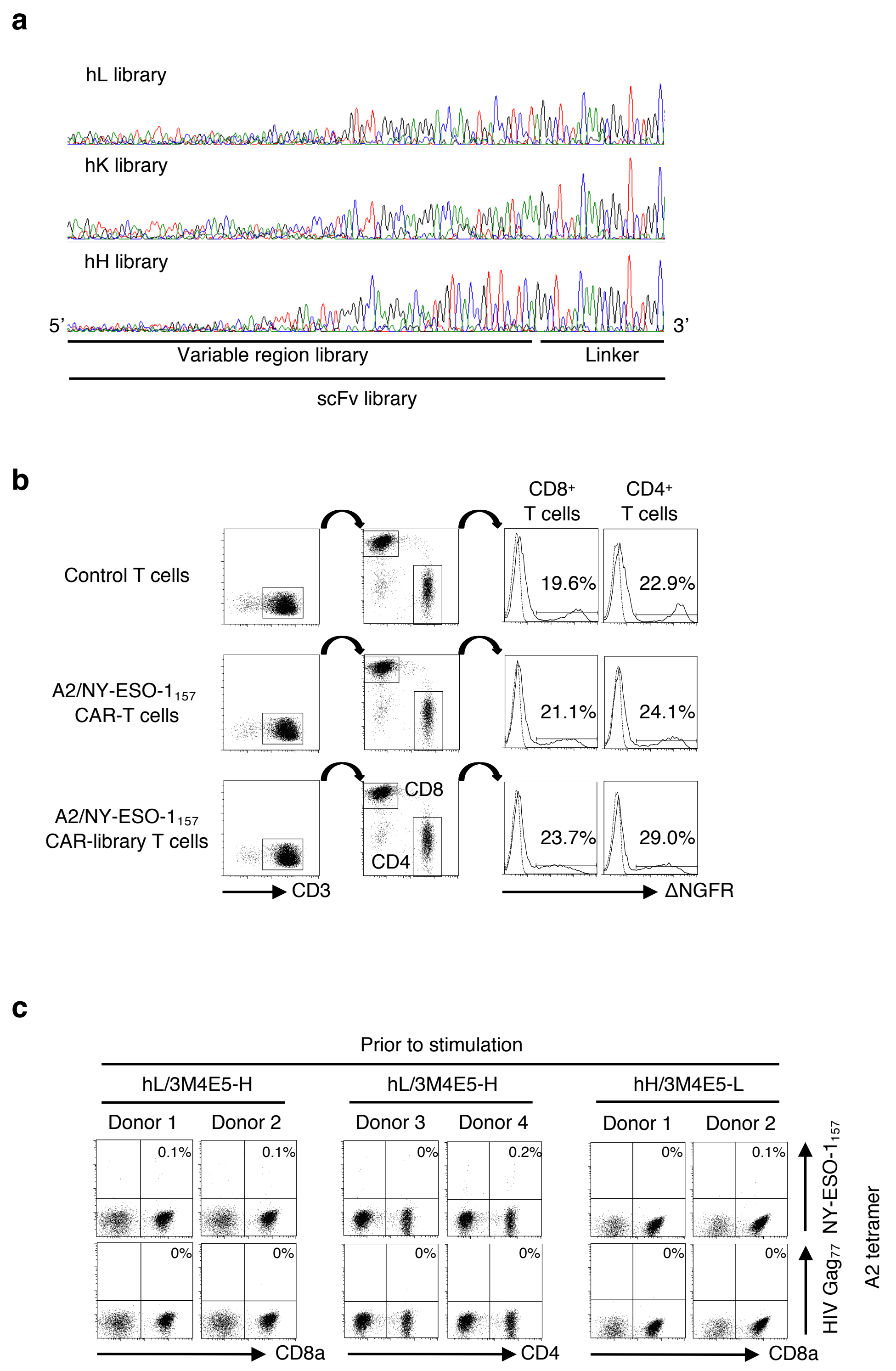

Supplementary Figure 2. Generation of A2/NY-ESO-1_{157} CAR-library T cells.

a An scFv library in which a variable region library from the lambda chain (hL), kappa chain (hK), or heavy chain (hH) was fused with 3M4E5-H or 3M4E5-L via a linker sequence. A representative chromatogram of each scFv library is shown. **b** Second-generation CAR encoding an scFv library followed by CD28ζ was transduced into peripheral blood T cells. Control T cells and the original A2/NY-ESO-1_{157} CAR-T cells (3M4E5-LH CAR-T cells) were prepared as a negative control and a positive control, respectively. ΔNGFR positivity in gene-modified $CD8^+$ T cells and $CD4^+$ T cells was measured by flow cytometry. The dotted line shows an isotype control. Representative dot plots and histograms are depicted. **c** A2/NY-ESO-1_{157} CAR-library T cells prior to stimulation were stained with 20 μg/mL A2/NY-ESO-1_{157} tetramer or A2/HIV Gag_{77} tetramer, and each dot plot is shown. Each percentage of A2/NY-ESO-1_{157} tetramer-positive cells among CAR-library T cells is shown. Stainings of the A2/NY-ESO-1_{157} CAR-library T cells after antigen-specific stimulation are depicted in Fig. 2a.

Supplementary Figure 3

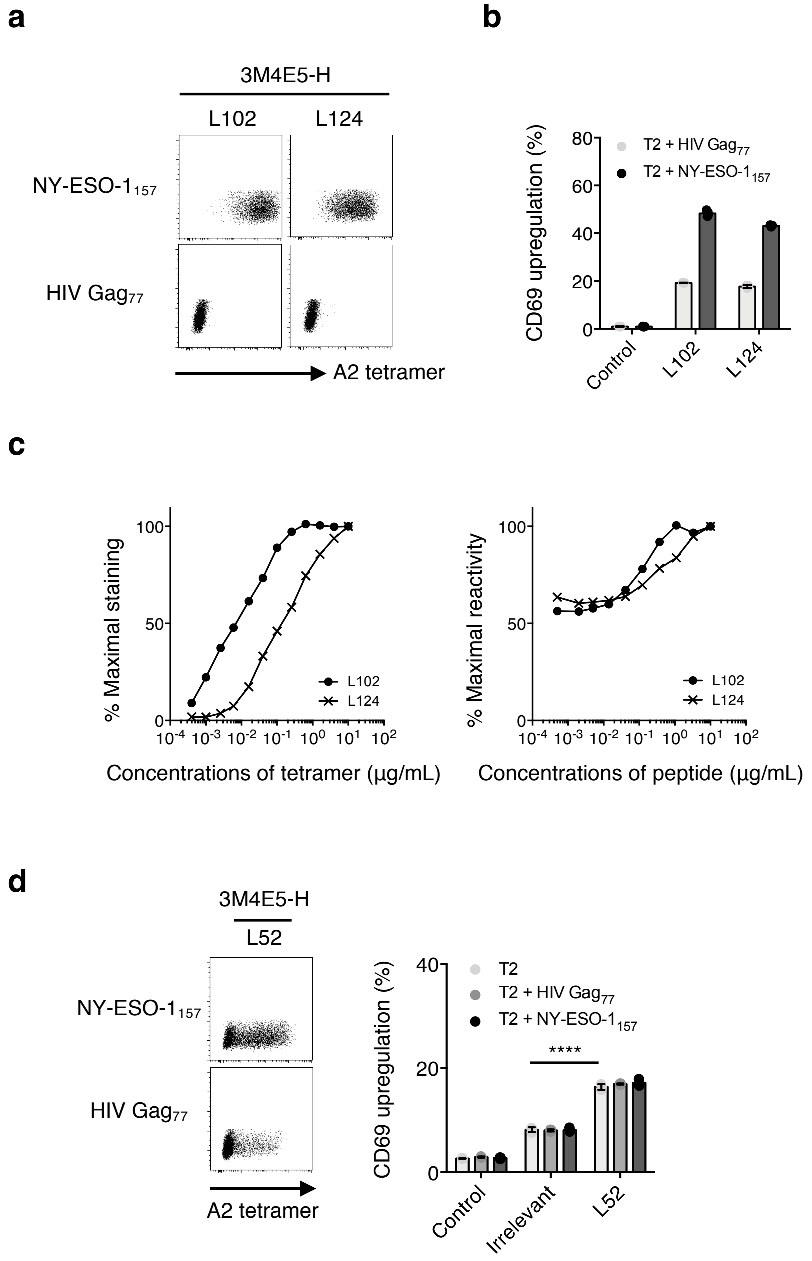

Supplementary Figure 3. Characteristics of new scFv-expressing A2/NY-ESO-1_{157} CAR-T cells.

a CAR encoding a new light chain (L102, or L124) paired with 3M4E5-H was individually reconstituted in Jurkat 76 cells. Jurkat 76/CAR transfectants were stained with 5 μg/mL A2/NY-ESO-1_{157} tetramer or A2/HIV Gag_{77} tetramer. **b** Jurkat 76/CAR transfectants were incubated with T2 cells pulsed with 10 μg/mL NY-ESO-1_{157} peptide or HIV Gag_{77} peptide. CD69 upregulation of the transfectants was measured by flow cytometry. The experiments were performed in triplicate, and error bars show the SD. **c** Jurkat 76/CAR transfectants were stained with graded concentrations of A2/NY-ESO-1_{157} tetramer (left). They were also incubated with T2 cells pulsed with graded concentrations of A2/NY-ESO-1_{157} peptide (right). Percentage maximal staining and percentage maximal reactivity of each transfectant was calculated, similarly to Fig. 2f. **d** The Jurkat 76/CAR transfectant expressing clone L52 paired with 3M4E5-H as the scFv was stained with 5 μg/mL A2/NY-ESO-1_{157} or A2/HIV Gag_{77} tetramer. CD69 upregulation of L52 CAR-T cells, irrelevant CAR-T cells, and control T cells was also measured by flow cytometry. The experiments were performed in triplicate, and error bars show the SD. Welch's t test (two-sided) was performed for comparison. ****, $p < 0.0001$.

Supplementary Figure 4

a

Peptide	Sequence	Binding affinity (nM)
NY-ESO-1$_{157\text{-}165}$	SLLMWITQC	666.69
LYPLAL1$_{36\text{-}44}$	GLRMWIKQV	1859.63

b

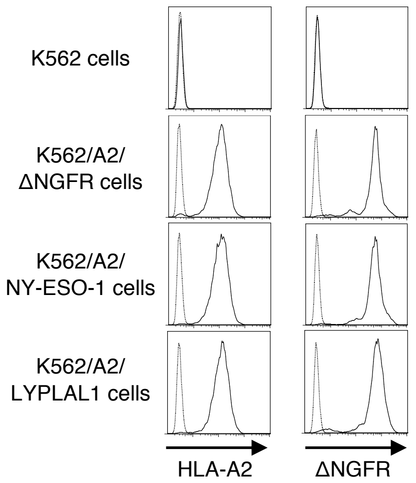

c

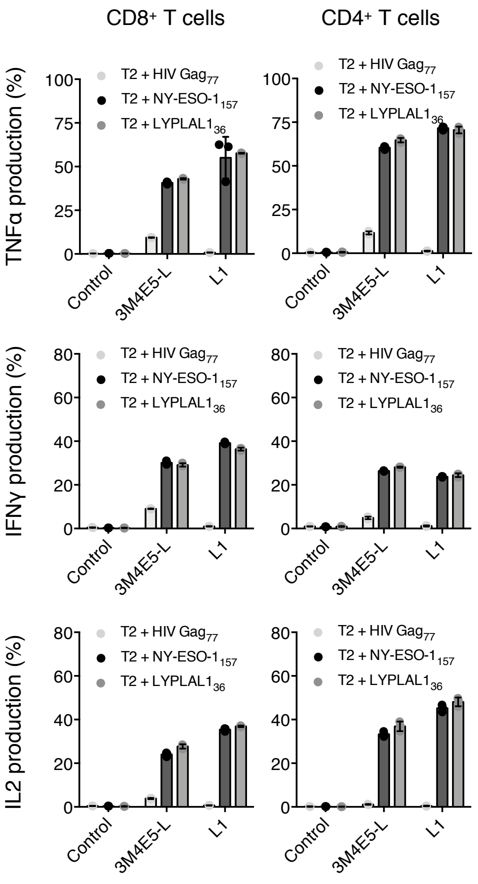

d

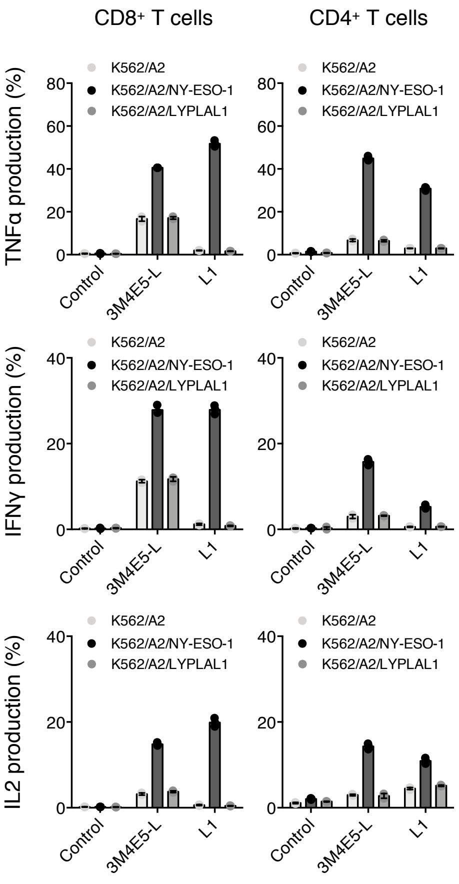

Supplementary Figure 4. Assessment of potential cross-reactivity mediated by L1 CAR-T cells. **a** Based on the peptide sequence motif (xLxMWIxQx) revealed by alanine scanning assays, one potential cross-reactive peptide, LYPLAL1_{36-44}, was identified. The amino acid sequences of NY-ESO-1_{157} and LYPLAL1_{36} peptides, and their predicted binding capacity to HLA-A*02:01 using netMHC4.0 (http://www.cbs.dtu.dk/services/NetMHC/) are also shown. **b** HLA-A2 and ΔNGFR expressions of K562 cells, K562/A2 cells, K562/A2/NY-ESO-1 cells, and K562/A2/LYPLAL1 cells were assessed by flow cytometry. The *ΔNGFR* gene was employed as a tag gene, as described in Methods. The dotted line shows an isotype control. **c, d** L1 CAR-T cells and 3M4E5-L CAR-T cells were incubated with T2 cells loaded with 10 μg/mL NY-ESO-1_{157} peptide, LYPLAL1_{36} peptide, or HIV Gag_{77} peptide (**c**). Then, these CAR-T cells were cocultured with K562-based target cells (**d**). Cytokine production by CAR-T cells was assessed using intracellular cytokine assays. The experiments were performed in triplicate, and error bars demonstrate the SD. The control T cells and CAR-T cells were prepared and analyzed similarly to Fig. 3.

Supplementary Figure 5

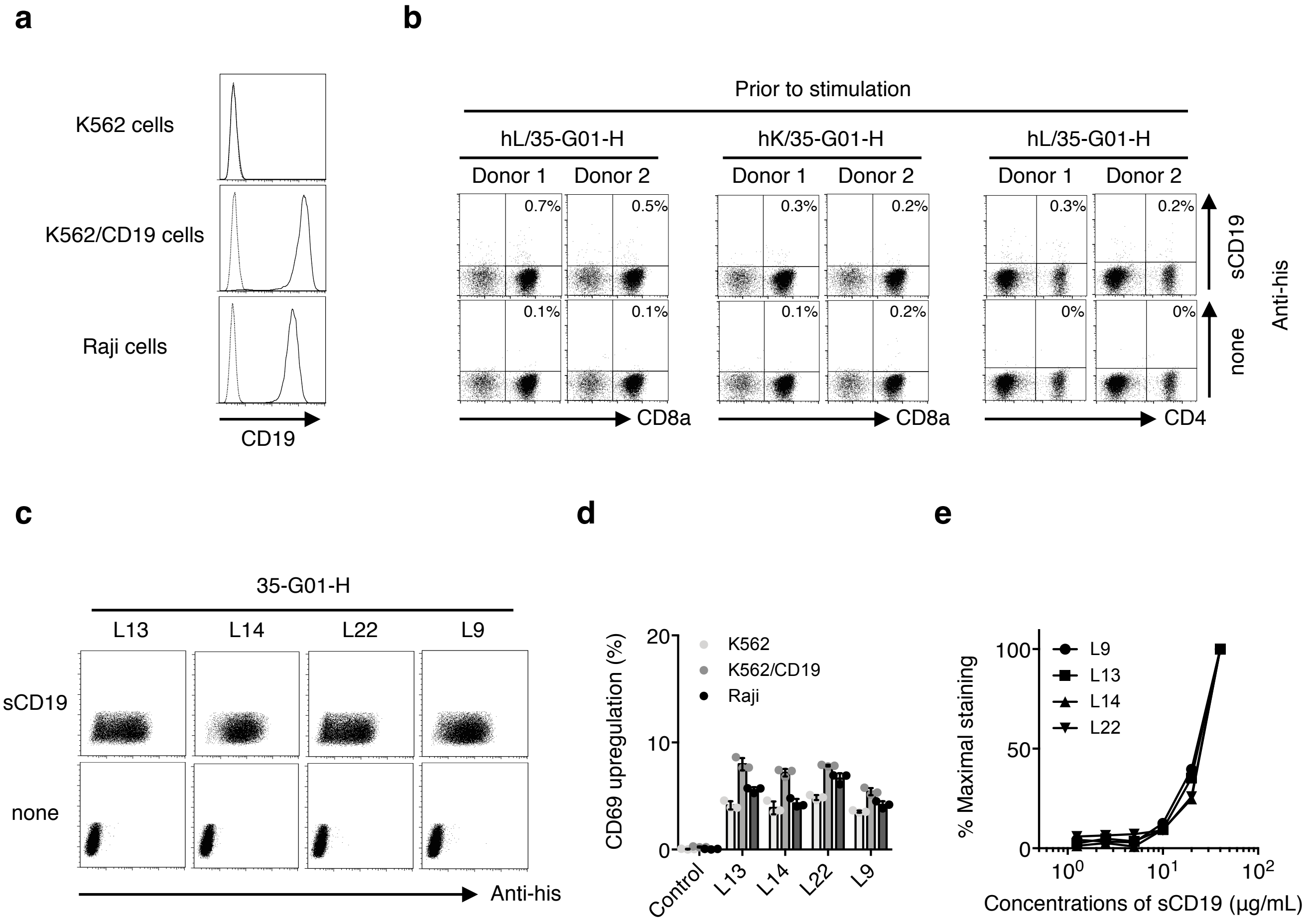

Supplementary Figure 5. Characteristics of new scFv-expressing CD19 CAR-T cells.

a CD19 expression of K562 cells, K562/CD19 cells, and Raji cells was assessed by flow cytometry. The dotted line shows an isotype control. **b** CD19 CAR-library T cells prior to stimulation were stained with 40 μg/mL sCD19 dimer or PE-anti-his mAb alone, and each dot plot is shown. Each percentage of sCD19 dimer-positive cells among CAR-library T cells is displayed. Stainings of the CD19 CAR-library T cells after antigen-specific stimulation are depicted in Fig. 4c. **c** CAR encoding newly isolated light chains (L13, L14, L22, or L9) paired with 35-G01-H was individually reconstituted in Jurkat 76 cells. Jurkat 76/CAR transfectants were stained with 40 μg/mL sCD19 dimer or PE-anti-his mAb alone. **d** Jurkat 76/CAR transfectants were incubated with the indicated target cells, and their CD69 upregulation was examined by flow cytometry. The experiments were performed in triplicate, and error bars show the SD. **e** Jurkat 76/CAR transfectants were stained with graded concentrations of sCD19 dimer. Percentage maximal staining was calculated, similarly to Fig. 4g.

Supplementary Figure 6

a

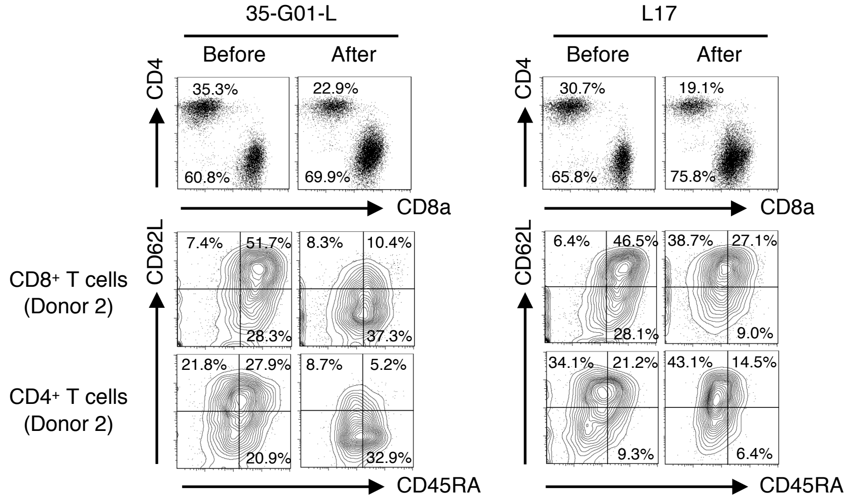

b

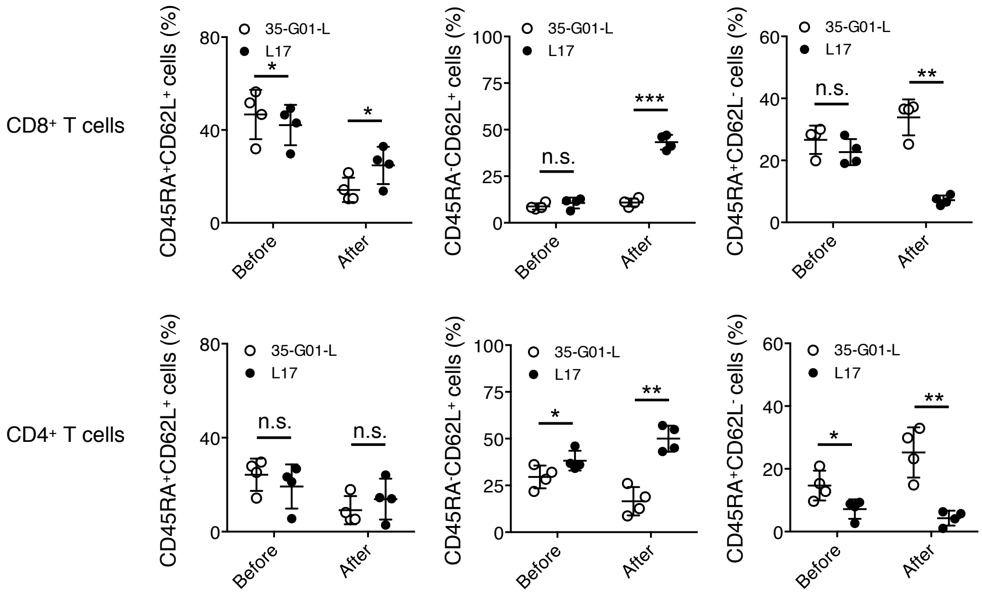

Supplementary Figure 6. T-cell phenotypes of CD19 CAR-T cells after stimulation.

a The positivity of $CD8^+$ and $CD4^+$ T cells among L17, or 35-G01-L CAR-T cells derived from donor 2 before and after stimulation with Raji cells is shown (top). The proportions of CD45RA and/or CD62L-positive cells among L17, or 35-G01-L CAR $CD8^+$ T cells and $CD4^+$ T cells (donor 2) are also depicted, respectively (middle, bottom). **b** The percentages of $CD45RA^+CD62L^+$, $CD45RA^-CD62L^+$, and $CD45RA^+CD62L^-$ cells among CAR $CD8^+$ T cells and $CD4^+$ T cells of 4 different donors before and after stimulation are shown. Each dot depicts the percentage of the indicated population among CAR-T cells generated from each donor. Differences between L17 and 35-G01-L CAR-T cells were statistically analyzed by paired t test (two-sided). *, $p < 0.05$; **, $p < 0.01$; ***, $p < 0.001$; n.s., not significant.

Supplementary Figure 7

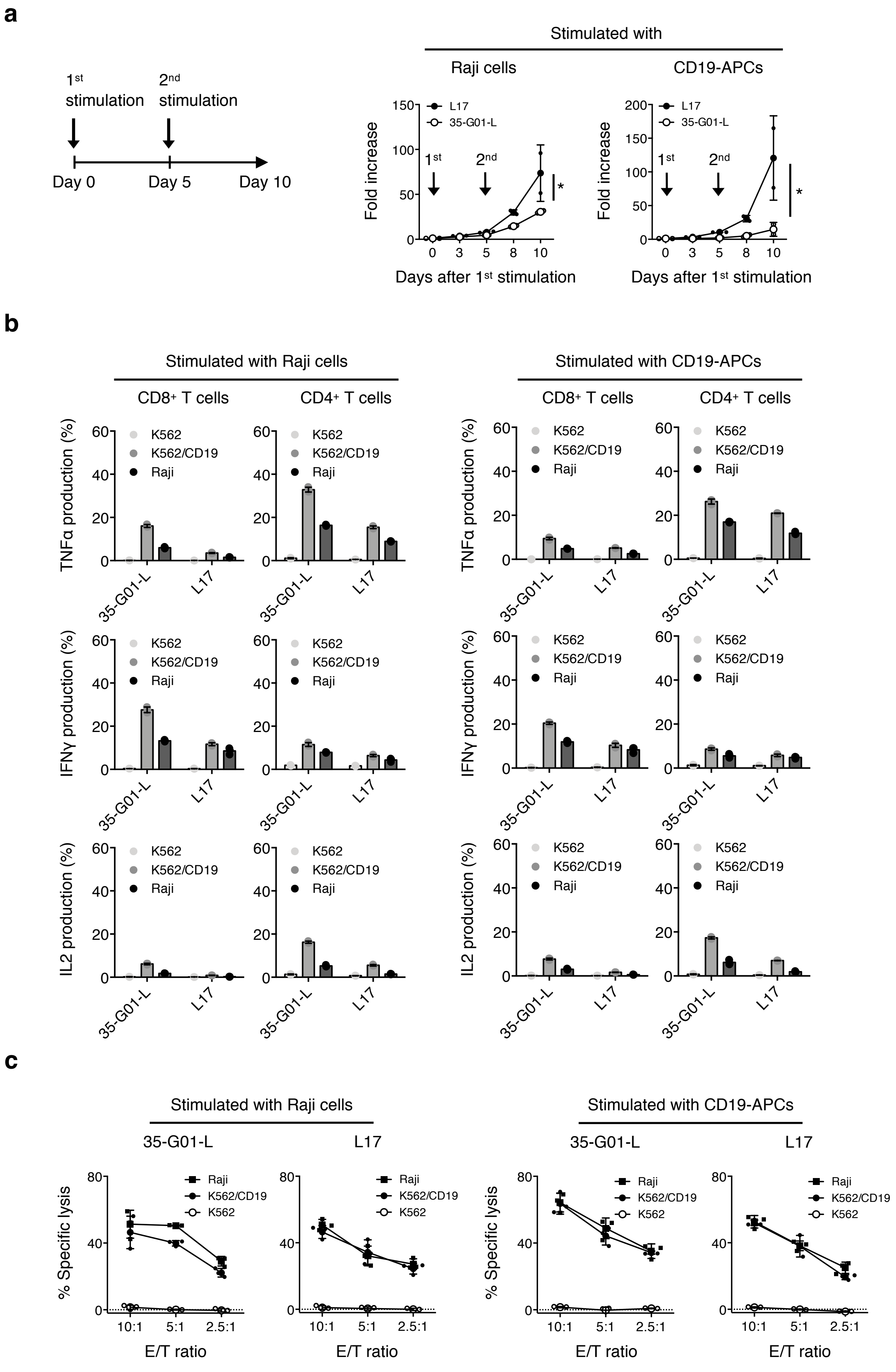

Supplementary Figure 7. Characteristics of CD19 CAR-T cells repeatedly stimulated with $CD19^{+}$ target cells.

a L17 and 35-G01-L CAR-T cells were stimulated twice with Raji cells (E/T 1:1) or CD19-APCs (E/T 10:1) on Day 0 and Day 5 (left). The fold increase of CAR-T cells established from 2 different donors after the first stimulation is shown (right). Arrows inside the graphs indicate the time point of stimulation. Error bars indicate the SD. Two-way ANOVA with Sidak's correction was conducted for multiple comparisons. *, $p < 0.05$. **b** Cytokine production by L17, and 35-G01-L CAR-T cells against the indicated target cells following two stimulations was assessed using intracellular cytokine assays. The experiments were performed in triplicate, and error bars show the SD. **c** Cytotoxicity of expanded L17 and 35-G01-L CAR-T cells against target cells was examined by ^{51}Cr-release assays. The experiments were performed in triplicate, and error bars depict the SD.

Supplementary Figure 8

a

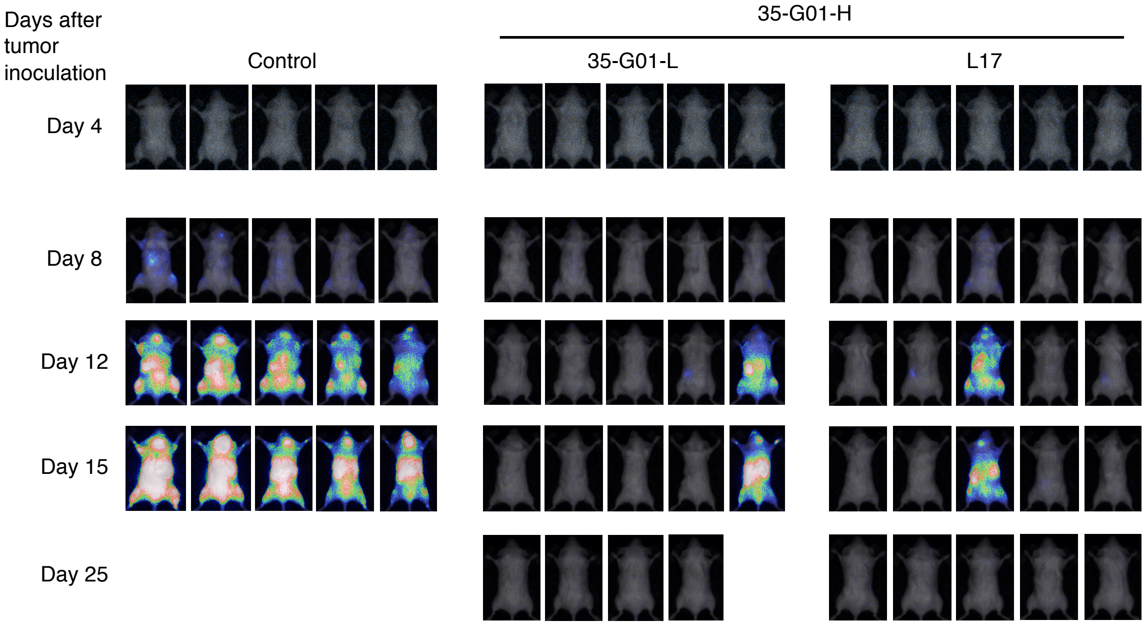

b

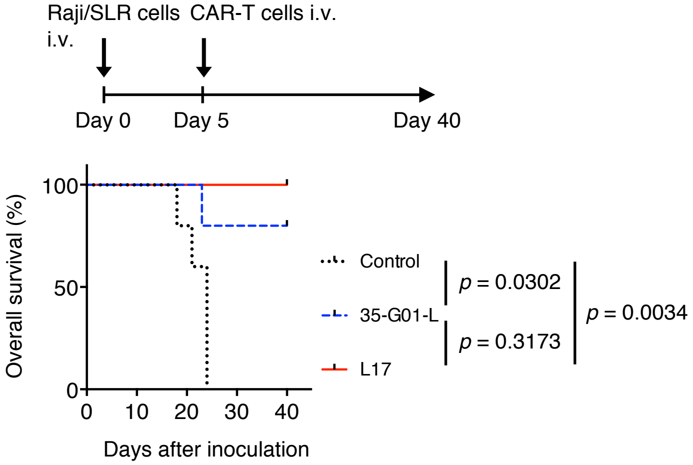

Supplementary Figure 8. *In vivo* antitumor effects of CD19 CAR-T cells.

a, b Five hundred thousand Raji/SLR cells were intravenously injected into irradiated NOD/Shi-scid IL2rgamma(null) mice. After confirming the engraftment of Raji/SLR cells by bioluminescence imaging assays on Day 4, $2.0x10^6$ CAR-T cells or control T cells were intravenously injected on Day 5. Photon images of mice on Days 8, 12, 15, and 25 are shown (**a**). The treatment schedule is summarized, and the overall survival of each group (n = 5 mice per group) is represented and compared in the form of a Kaplan-Meier curve, similarly to Fig. 6a. Log-rank (Mantel-Cox) test was performed, and *p* values are also shown (**b**).

Supplementary Figure 9

a

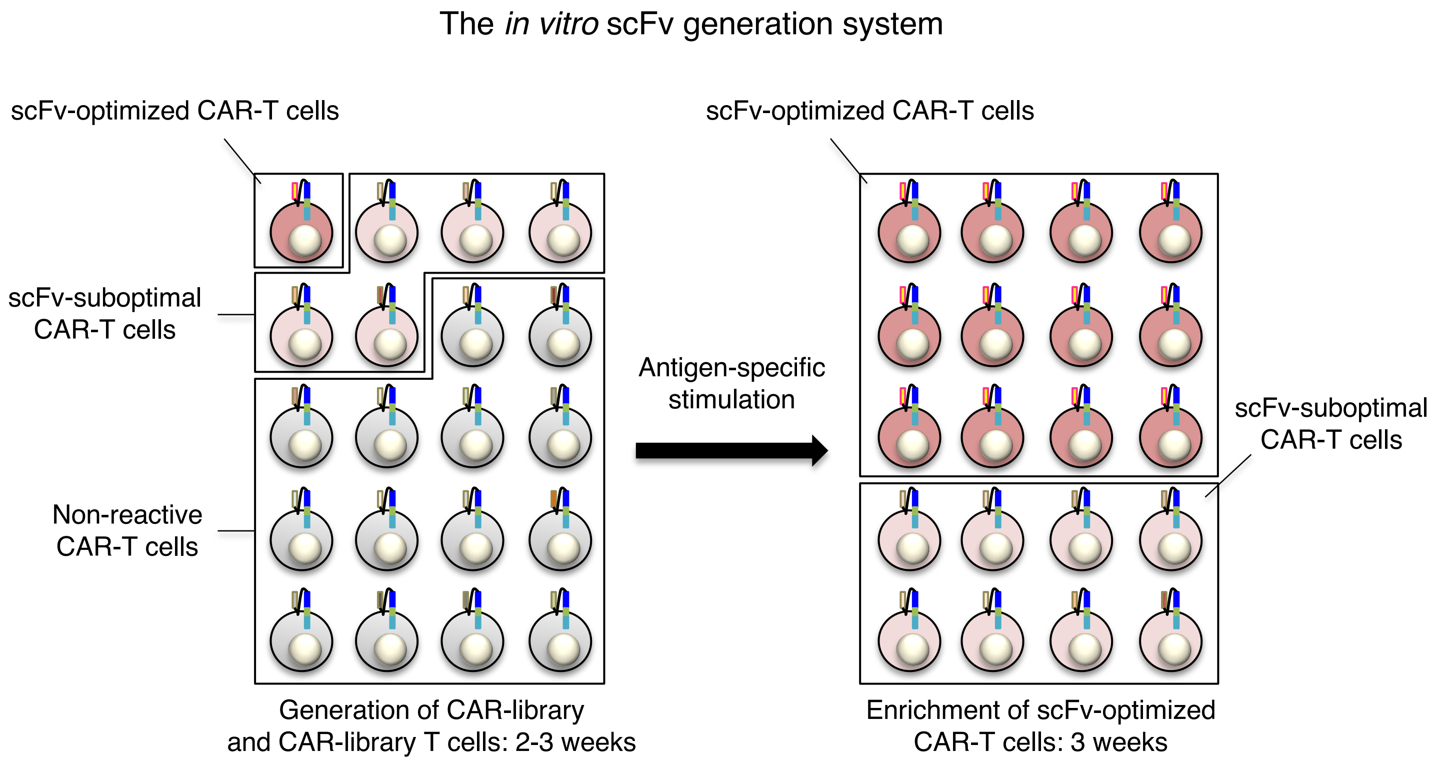

b

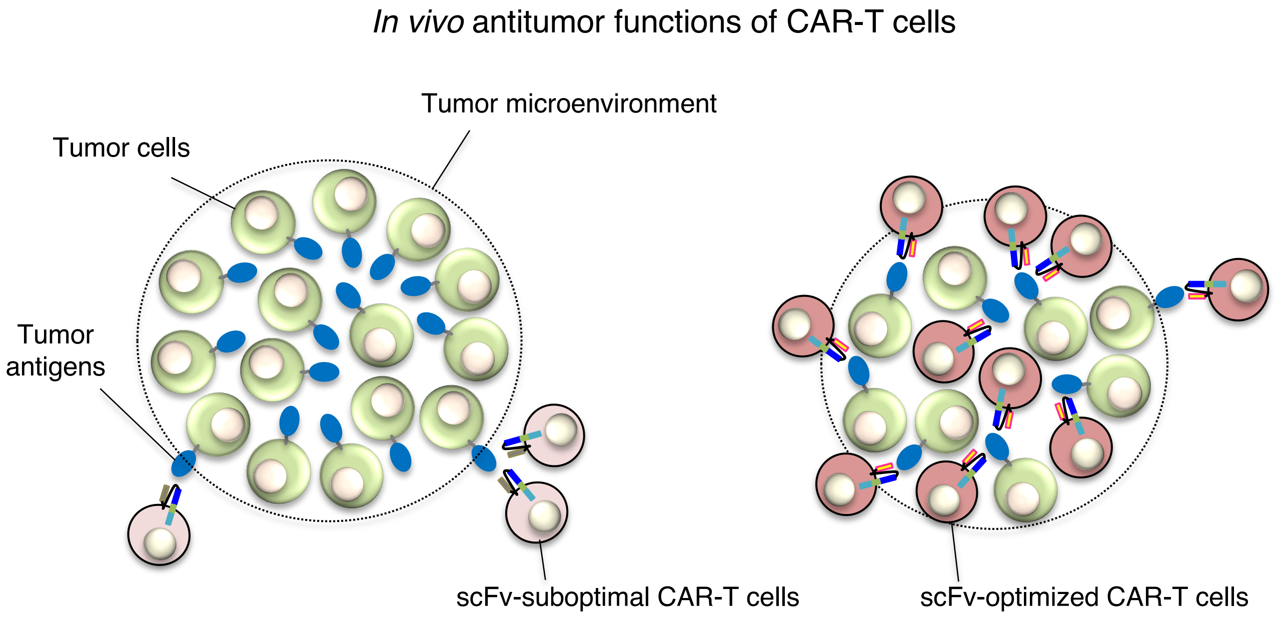

Supplementary Figure 9. The *in vitro* scFv generation system potentially yielding CAR-T cells with superior *in vivo* antitumor function.

a The *in vitro* scFv generation system using CAR-library T cells. ScFv-optimized (dark red), scFv-suboptimal (light red), and non-reactive (gray) CAR-T cells are included among CAR-library T cells (left). Since scFv-optimized CAR-T cells have a survival and proliferation advantage over scFv-suboptimal CAR-T cells after antigen-specific stimulation of CAR-library T cells, the former appears to be well concentrated, allowing identification of new scFvs that can endow CAR-T cells with superior antitumor function *in vivo* (right). The times required for generation of CAR-library and CAR-library T cells and enrichment of scFv-optimized CAR-T cells are shown similarly to Fig. 1. The supporting data are shown in Fig. 5, Supplementary Fig. 6, 7. **b** The cartoons summarize the results shown in Fig. 6. ScFv-suboptimal CAR-T cells (light red) appear to be localized around the tumor site *in vivo* (left), whereas scFv-optimized CAR-T cells (dark red) seem to infiltrate diffusely and accumulate within the tumor, recognizing and killing tumor cells *in vivo* (right). These *in vivo* antitumor effects may be explained by sufficient antitumor cytotoxicity, superior proliferation capacity, and durable phenotypes of CAR-T cells expressing the scFv optimized by our *in vitro* scFv generation system.

Supplementary Table 1

Clone	Variable region	CDR3	J region	Structural avidity (EC_{50}: µg/mL)	Functional avidity (EC_{50}: µg/mL)
**Variable regions of light chain (lambda chain) pairing with 3M4E5-H**					
3M4E5-L	2-11*01	CWSFAGSYYVF	1*01	0.002	0.162
L1	2-8*01	CSSYAGSNSVF	1*01	0.005	0.031
L102	2-8*01	CSSYAGSGSTPFVF	1*01	0.017	0.122
L73	2-14*01	CSSYSGSSTWVF	3*02	0.036	0.270
L124	2-14*01	CCSYAGRRYVF	1*01	0.141	0.624
L88	2-11*01	CCSYAGGYYVF	1*01	0.171	0.459
L66	2-14*01	CSSYTSSSTYVF	1*01	1.197	0.997
L80	2-14*02	CSSYTSSSTFAVF	7*01	NC	NC
L52	1-40*01	CGTWDSSLSAGVF	7*01	NC	NC
**Variable regions of heavy chain pairing with 3M4E5-L**					
3M4E5-H	3-23*01	CAGELLPYYGMDVW	6*02	0.009	0.248
H73	4-34*09	CARCPIYYYGMDVW	6*02	0.016	0.043
H1	4-59*01	CARESYYYYGMDVW	6*02	0.066	0.065

Supplementary Table 1. Variable regions and CDR3 sequences of A2/NY-ESO-1_{157}-specific VL and VH, and characteristics of CAR-T cells expressing each scFv.

According to the nomenclature of the IMGT (http://www.imgt.org/), the variable regions and amino acid sequences of the CDR3 region of newly identified and original light chains and heavy chains specific for A2/NY-ESO-1_{157} are summarized. The structural avidity and functional avidity of Jurkat 76/CAR-T cells expressing each scFv are shown as the EC_{50} value (μg/mL), measured as described in Fig. 2. NC, not calculated.

Supplementary Table 2

Clone	Variable region	CDR3	J region	Structural avidity (EC_{50}: μg/mL)
**Variable regions of light chain (lambda chain) pairing with 35-G01-H**				
35-G01-L	2-8*01	CCSYAGRYNSVLF	2*01	9.92
L17	1-47*01	CAAWDDSLSAIF	2*01	11.98
L16	2-8*01	CAAWDDSLNGVVF	2*01	16.28
L7	2-11*01	CGTWDTSLTAVVF	2*01	17.93
L9	1-51*01	CGTWESSLSGVVF	2*01	18.84
L13	2-14*01	CSSYTTSTTWVF	3*02	19.23
L14	2-14*01	CCSYAGSYTFVVF	2*01	20.91
L22	2-14*01	CHSYDSSLSHVF	1*01	21.31
L4	3-1*01	CQAWDSSTHVVF	2*01	21.58
**Variable regions of light chain (kappa chain) pairing with 35-G01-H**				
K5	3-11*01	CQQSYSTLLYTF	2*01	12.97
K4	3-15*01	CQQYNNWPPLYTF	2*01	13.37
K9	3-20*02	CQQFNEWPLTF	4*01	15.38
K6	3-15*01	CQQYDSLPLTF	4*01	18.01

Supplementary Table 2. Variable regions and CDR3 sequences of CD19-specific VL, and characteristics of CAR-T cells expressing each scFv.

Based on the nomenclature of the IMGT (http://www.imgt.org/), the variable regions and amino acid sequences of the CDR3 region of newly isolated and original light chains specific for CD19 are listed. The structural avidity of CAR-T cells possessing each scFv is shown as the EC_{50} value (μg/mL), calculated as the concentration of sCD19 dimer required to achieve 50% of the maximal staining.
