# Supplementary material for: A single-chain antibody generation system yielding CAR-T cells with superior antitumor function
Source: Commun Biol. 2021 Mar 2;4:273. doi: 10.1038/s42003-021-01791-1 (PMC7925539; doi:10.1038/s42003-021-01791-1)
Supplement: Supplementary file 2 — Description of Additional Supplementary Files [file 42003_2021_1791_MOESM2_ESM.pdf]

Description of Additional Supplementary Files

File Name: Supplementary Data 1

Description: Source data for graphs in the main figures.
