# Supplementary material for: A single-chain antibody generation system yielding CAR-T cells with superior antitumor function
Source: Commun Biol. 2021 Mar 2;4:273. doi: 10.1038/s42003-021-01791-1 (PMC7925539; doi:10.1038/s42003-021-01791-1)
Supplement: Supplementary file 4 — Reporting Summary [file 42003_2021_1791_MOESM4_ESM.pdf]

nature research

Corresponding author(s): Toshiki Ochi

Last updated by author(s): 2020/12/5

Reporting Summary

Nature Research wishes to improve the reproducibility of the work that we publish. This form provides structure for consistency and transparency in reporting. For further information on Nature Research policies, see our Editorial Policies and the Editorial Policy Checklist.

Statistics

For all statistical analyses, confirm that the following items are present in the figure legend, table legend, main text, or Methods section.

n/a	Confirmed	
☐	☒	The exact sample size (*n*) for each experimental group/condition, given as a discrete number and unit of measurement
☐	☒	A statement on whether measurements were taken from distinct samples or whether the same sample was measured repeatedly
☐	☒	The statistical test(s) used AND whether they are one- or two-sided<br>*Only common tests should be described solely by name; describe more complex techniques in the Methods section.*
☐	☒	A description of all covariates tested
☐	☒	A description of any assumptions or corrections, such as tests of normality and adjustment for multiple comparisons
☐	☒	A full description of the statistical parameters including central tendency (e.g. means) or other basic estimates (e.g. regression coefficient) AND variation (e.g. standard deviation) or associated estimates of uncertainty (e.g. confidence intervals)
☐	☒	For null hypothesis testing, the test statistic (e.g. *F*, *t*, *r*) with confidence intervals, effect sizes, degrees of freedom and *P* value noted<br>*Give P values as exact values whenever suitable.*
☒	☐	For Bayesian analysis, information on the choice of priors and Markov chain Monte Carlo settings
☒	☐	For hierarchical and complex designs, identification of the appropriate level for tests and full reporting of outcomes
☐	☒	Estimates of effect sizes (e.g. Cohen's *d*, Pearson's *r*), indicating how they were calculated

Our web collection on statistics for biologists contains articles on many of the points above.

Software and code

Policy information about availability of computer code

Data collection	No software was used.
Data analysis	Prism v6.0h (GraphPad), FlowJo v7.6.5 (TreeStar), Fiji v1.0

For manuscripts utilizing custom algorithms or software that are central to the research but not yet described in published literature, software must be made available to editors and reviewers. We strongly encourage code deposition in a community repository (e.g. GitHub). See the Nature Research guidelines for submitting code & software for further information.

Data

Policy information about availability of data

All manuscripts must include a data availability statement. This statement should provide the following information, where applicable:
- Accession codes, unique identifiers, or web links for publicly available datasets
- A list of figures that have associated raw data
- A description of any restrictions on data availability

Source data for graphs in the main figures are available in the Supplementary Data 1. All data supporting the conclusions of this study are included in the manuscript and its supplementary files, or are available from the corresponding author upon reasonable request.

Field-specific reporting

Life sciences study design

All studies must disclose on these points even when the disclosure is negative.

Sample size	Sample sizes were determined based on the preliminary experiments.
Data exclusions	No data were excluded from the analyses.
Replication	All attempts at replication were successful.
Randomization	Mice were allocated into each experimental group not to yield any differences in each group prior to treatment.
Blinding	No blinded analyses were performed.

Reporting for specific materials, systems and methods

We require information from authors about some types of materials, experimental systems and methods used in many studies. Here, indicate whether each material, system or method listed is relevant to your study. If you are not sure if a list item applies to your research, read the appropriate section before selecting a response.

Materials & experimental systems

n/a	Involved in the study	
☐	☒	Antibodies
☐	☒	Eukaryotic cell lines
☒	☐	Palaeontology and archaeology
☐	☒	Animals and other organisms
☒	☐	Human research participants
☒	☐	Clinical data
☒	☐	Dual use research of concern

Methods

n/a	Involved in the study	
☒	☐	ChIP-seq
☐	☒	Flow cytometry
☒	☐	MRI-based neuroimaging

Antibodies

Antibodies used

1. Anti-human CD3 mAb (clone OKT3, Biolegend) for T-cell stimulation.

2. FITC-anti-human NGFR mAb (clone ME20.4, Biolegend) for isolation of gene-modified cells.

3. PC5-anti-human CD8 mAb (clone B9.11, Beckman Coulter), FITC-anti-human CD4 mAb (clone OKT4, Biolegend), V450-anti-human NGFR mAb (clone C40-1457, Becton Dickinson), APC-anti-human CD3 mAb (clone UCHT1, Biolegend), Biotinylated HLA-A2/NY-ESO-1_157 monomer (MBL), Biotinylated HLA-A2/HIV Gag_77 monomer (MBL), PE-streptavidin (Thermo Fisher Scientific), PE-anti-His mAb (clone GG11-8F3.5.1, Miltenyi), PE-anti-HLA-A2 mAb (clone BB7.2, Biolegend), PE-anti-human NGFR mAb (clone ME20.4, Biolegend), PE-anti-human CD19 mAb (clone HIB19, Biolegend), PE-anti-human TNFa mAb (clone MAb11, Biolegend), APC-anti-human IL2 mAb (clone MQ1-17H12, Biolegend), PC7-anti-human IFNg mAb (clone B27, Biolegend), PE-anti-human PD1 mAb (clone EH12.2H7, Biolegend), APC-anti-human CD45RA mAb (clone HI100, Biolegend), PC7-anti-human CCR7 mAb (clone G043H7, Biolegend), BV421-anti-human CD62L mAb (clone DREG-56, Biolegend), APC-Cy7-anti-human CD4 mAb (clone RPA-T4, Biolegend), FITC-anti-human CD69 mAb (clone FN50, Biolegend) for flow cytometry.

4. Anti-human CD20 mAb (clone L26, Abcam), anti-human CD8 mAb (clone C8/144B, Abcam), anti-human CD4 mAb (clone 4B12, Thermo Fisher Scientific) for immunohistochemistry.

Validation

Validation of each primary antibody was performed by the manufacturer, and data are available on the manufacturer's website.

Eukaryotic cell lines

Policy information about cell lines

Cell line source(s)	K562, T2, Raji, and PG13 were purchased from ATCC. Plat-A was kindly provided by Dr. Toshio Kitamura (Institute of Medical Sciences, University of Tokyo). Jurkat 76 was a generous gift from Dr. Mirjam Heemskerk (Leiden University).
Authentication	None of the cell lines used were authenticated.
Mycoplasma contamination	All cell lines tested were negative for mycoplasma contamination.
Commonly misidentified lines (See ICLAC register)	None.

Animals and other organisms

Policy information about studies involving animals; ARRIVE guidelines recommended for reporting animal research

Laboratory animals	NOG mice (NOD/Shi-scid IL2rgamma(null), In-Vivo Science Inc.); female; 5-week old.
Wild animals	The study did not involve wild animals.
Field-collected samples	The study did not involve samples collected from the field.
Ethics oversight	All the murine experiments in this study were approved by the Ehime University Animal Care Committee.

Note that full information on the approval of the study protocol must also be provided in the manuscript.

Flow Cytometry

Plots

Confirm that:

- [x] The axis labels state the marker and fluorochrome used (e.g. CD4-FITC).
- [ ] The axis scales are clearly visible. Include numbers along axes only for bottom left plot of group (a 'group' is an analysis of identical markers).
- [ ] All plots are contour plots with outliers or pseudocolor plots.
- [x] A numerical value for number of cells or percentage (with statistics) is provided.

Methodology

Sample preparation	The cells were suspended with FACS buffer (2%FCS/PBS), and stained with a cocktail of fluorochrome-conjugated antibodies. Dead cells were also stained with LIVE/DEAD Fixable Near-IR Dead Cell Stain Kit (Thermo Fisher Scientific). Stained cells were washed twice with FACS buffer and then analyzed.
Instrument	Gallios flow cytometer (Beckman Coulter).
Software	FlowJo v7.6.5.
Cell population abundance	Ten to one hundred thousand events in FSC/SSC parameters were measured to analyze.
Gating strategy	The Near-IR negative cells were gated using FSC and SSC parameters. Truncated NGFR tag-positive T cells were gated to analyze when stained with anti-human NGFR mAb.

- [x] Tick this box to confirm that a figure exemplifying the gating strategy is provided in the Supplementary Information.
